# Anti-Colonization Effect of Au Surfaces with Self-Assembled Molecular Monolayers Functionalized with Antimicrobial Peptides on *S. epidermidis*

**DOI:** 10.3390/antibiotics10121516

**Published:** 2021-12-10

**Authors:** Eskil André Karlsen, Wenche Stensen, Eric Juskewitz, Johan Svenson, Mattias Berglin, John Sigurd Mjøen Svendsen

**Affiliations:** 1 Amicoat AS, Sykehusvegen 23, 9019 Tromsø, Norway; eskil.a.karlsen@uit.no (E.A.K.); wenche.stensen@uit.no (W.S.); 2 Department of Chemistry, Faculty of Science and Technology, UiT—The Arctic University of Norway, 9037 Tromsø, Norway; 3 Department of Medical Biology, Faculty of Health Sciences, UiT—The Arctic University of Norway, 9037 Tromsø, Norway; eric.juskewitz@uit.no; 4 RISE Research Institutes of Sweden, Brinellgatan 4, 504 62 Borås, Sweden; johan.svenson@cawthron.org.nz (J.S.); mattias.berglin@ri.se (M.B.)

**Keywords:** antimicrobial surface, antimicrobial peptide, self-assembled monolayer, antifouling, anti-colonization, ToF-SIMS imaging, Certika

## Abstract

Medical devices with an effective anti-colonization surface are important tools for combatting healthcare-associated infections. Here, we investigated the anti-colonization efficacy of antimicrobial peptides covalently attached to a gold model surface. The gold surface was modified by a self-assembled polyethylene glycol monolayer with an acetylene terminus. The peptides were covalently connected to the surface through a copper-catalyzed [3 + 2] azide-acetylene coupling (CuAAC). The anti-colonization efficacy of the surfaces varied as a function of the antimicrobial activity of the peptides, and very effective surfaces could be prepared with a 6 log unit reduction in bacterial colonization.

## 1. Introduction

In the rich parts of the world, healthcare-associated infections (HAI) hit 1 of 14 hospitalized patients, often requiring additional treatment and extended hospitalization [1]. The highest frequency of HAI is associated with the use of invasive devices, in particular central lines, urinary catheters, and ventilators [2]. A device-associated infection accounts for up to 23% out of all healthcare-associated infections, including central line-associated bloodstream infections (CLABSI), catheter-associated urinary tract infections (CAUTI), and ventilator-associated pneumonia [2]. There is a goal to limit the number of HAIs, and in 2015, the U.S. Department of Health and Human Services set a target to reduce the infection rate by 25–50% by 2020 through stricter regulations and guidelines regarding the use of the devices [3,4]. Despite the progress of reducing CLABSIs and CAUTIs through best practice, the underlying problem remains—the surfaces of medical devices are still prone to bacterial growth and biofilm formation.

Bacteria can adhere irreversibly to living and non-living surfaces, colonize, and subsequently develop into an enclosed structured society of both Gram-positive and/or Gram-negative bacteria that adhere to a surface, forming a protective matrix known as a biofilm [5]. The most common bacteria found in biofilms are *E. faecalis*, *S. aureus*, *S. epidermidis*, *E. coli*, *K. pneumoniae*, and *P. aeruginosa* [6]. Biofilms are challenging to eradicate due to the extracellular polymeric material that creates a “slime-like” matrix, which acts as a protective barrier for the bacteria [5].

Indwelling medical devices (e.g., catheters) are particularly prone to bacterial colonization on the surface and subsequent biofilm formation, leading to infections in the patient [6]. Biofilm-associated infections on indwelling medical devices prolong initial treatment regimens, increase antibiotic use, and add additional healthcare expenses for the hospital and society. As an example, a central line-associated bloodstream infection can cost up to USD 46,000 per case and the patient can be hospitalized for an additional 7 days or more, depending on the type and severity of infection [7]. In addition, patients are at risk of sepsis and the mortality rate can be up to 25% [7]. A study conducted from 2015 to 2017 showed the most common bacteria reported on indwelling devices were *Escherichia coli* (18%), *Staphylococcus aureus* (12%), and *Klebsiella* spp. (9%) [8]. Another study conducted from 2008 to 2017 showed that 22% (48 out of 213 patients) of the device-associated infections were caused by multidrug-resistant bacteria [9].

If medical device-associated infections can be avoided, it would reduce antibiotic use and cost, and hence, contribute to alleviating the antimicrobial resistance crisis. Manufacturers of medical devices have worked intensely to create technologies that would diminish the infection risk originating from the use of such devices. Several techniques have been tried, and the use of heavy metals (like silver or copper in metal or ionic form [10,11,12]) or antibiotics (gentamicin, nitrofurazone, norfloxacin, minocycline-rifampicin, and more [13]) integrated into the devices, creating anti-colonization (antifouling) surfaces, are currently the most common solutions. However, limited efficacy, unwanted toxicity, and the non-degradability of silver and other heavy metals have kept the medical device industry looking for better alternatives [12,14,15]. Another issue with antibiotic-coated surfaces is the risk of contributing to the development of antibiotic resistance. As an example, the widespread use of triclosan as an antibacterial agent in medical devices and a variety of consumer products has not only triggered resistance against triclosan [16,17] but also against ciprofloxacin [18].

Coating the surface with covalently attached antimicrobial peptides (AMPs) can be an alternative solution to the problem of medical device-associated infections [19,20]. AMPs [21] have many advantages over the current antimicrobial agents used in medical devices. Antimicrobial peptides intrinsically biodegrade into amino acids, and hence, the problem with antibiotic persistence is limited. Furthermore, AMPs generally have negligible side effects, a broad action spectrum against both Gram-positive and Gram-negative bacteria, and they may even eradicate established biofilms [22,23,24]. The mechanism of action of AMPs is generally believed to involve microbial membrane destabilization or lysis [25], and hence, the risk of promoting resistance development is low compared to classic antibiotics that address a specific drug target [26]. An additional advantage of AMPs is the information of pharmacophores, that is, the minimum content of essential features, enabling a de novo design of synthetic peptides with pre-determined antibacterial activities [27]. AMPs and mimics of antimicrobial peptides have been shown to inhibit bacteria from colonizing on the surface either by releasing active agents or by covalently attaching to surfaces [20,28], although the surface immobilization of AMPs may lower the activity of the peptides [29].

**Figure 1:**
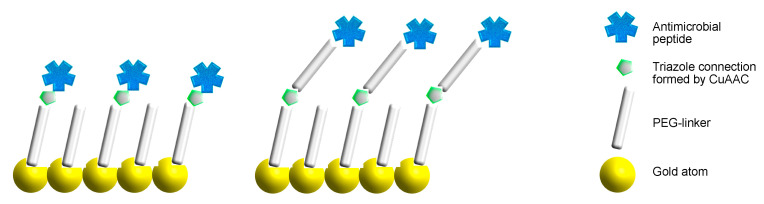
The design of the peptide-functionalized self-assembled monolayer (SAM) gold surface. The peptides were connected by a CuAAC reaction either directly to the terminus of the SAM (peptides **1c**, **1d**, **2c**, and **2d**, left), or through an additional PEG linker (peptides **1a**, **1b**, **2a**, and **2b**, right).

In the present study, the quantitative effect of antimicrobial peptides covalently attached to a surface upon the colonization of an avidly biofilm-forming bacterium, *Staphylococcus epidermidis*, was investigated. A gold surface with a polyethylene glycol (PEG) self-assembled molecular monolayer (SAM) was chosen as the model system since its preparation is a versatile and established technique [30]. A chemically addressable SAM monolayer composed of α-thio-PEG-ω-alkyne monolayers that spontaneously form on the Au (111) surface was used as the experimental platform because the peptides could be introduced as azide derivatives on the alkyne-molecular monolayer through a copper(I)-catalyzed alkyne-azide cycloaddition (CuAAC) reaction [31]. The use of a common surface where the peptides were covalently linked through the same chemical reaction allows for the preparation of a set of similar surfaces that differ only in the presence of an additional PEG linker and the type of peptide, and subsequently allows for the determination of a structure–activity relationship involving these features (Figure 1).

## 2. Results

### 2.1. Surface Design and Preparation

**Figure 2:**
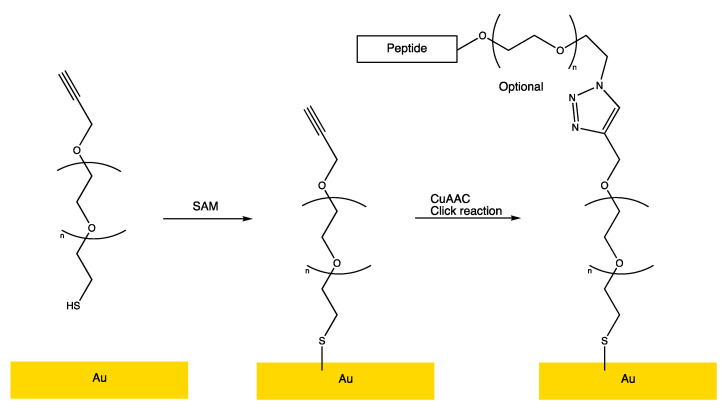
The preparation of the peptide-functionalized Au(–SAM) surface. A hydrophilic Au–SAM surface was made by reacting an Au(111) substrate with α-thio-PEG600-ω-alkyne (**left**→**middle** panel). The peptide-functionalized surface was created by a CuAAC reaction between the acetylene terminated Au–SAM surface and an azide-functionalized peptide (**middle**→**right** panel).

A quantitative investigation of the anti-colonization effect of antimicrobial peptides covalently attached to a model surface requires the restriction of uncontrolled parameters as much as possible. Hence, the functionalized surfaces were prepared using well-established and reliable methods. The peptides were connected to the surface through a Cu(I)-catalyzed [3 + 2] acetylene-azide cycloaddition (CuAAC, click) reaction between the surface-bound acetylene group and an azide functional group connected to the peptide, which is a reliable, high-yielding, and bioorthogonal method [31]. The selection of the CuAAC reaction for the peptide connection requires the surface to have an acetylene terminus. Hence, the Au–SAM surface was constructed through the self-assembly of α-thio-PEG600-ω-alkyne on an Au(111) substrate (Figure 2, left) through a self-assembled monolayer (Figure 2, middle), terminating in the required alkyne functional group. Furthermore, a PEG-based SAM surface was chosen to ensure the hydrophilic surface interacted well with the aqueous environment. The CuAAC-linking of the peptide effector molecules to the Au–SAM surface generates a stable covalent connection through a 1,2,3-triazole moiety (Figure 2, right) [32].

### 2.2. Peptide Design

**Figure 3:**
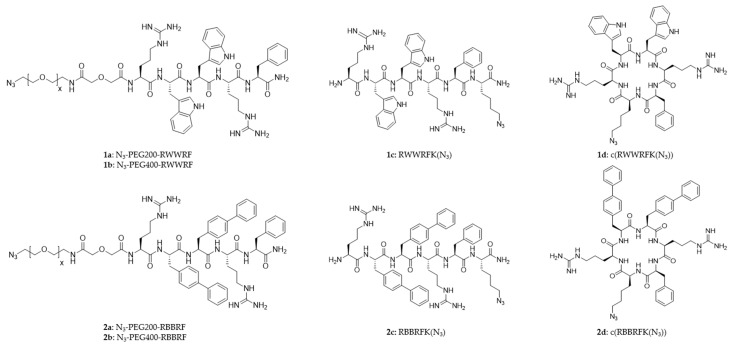
Eight azidopeptides with different hydrophobic groups (Series 1: tryptophan, Series 2: biphenylalanine).

The peptides were designed according to the pharmacophore of cationic antimicrobial peptides [27,33], with five or six residues containing two arginine residues and three bulky and lipophilic moieties in the form of tryptophan, phenylalanine, and 4′-phenyl-phenylalanine (biphenylalanine, B) [34,35]. Series 1 (**1a**–**d**) contains tryptophan as the major hydrophobic group, while Series 2 (**2a**–**d**) contains biphenylalanine as the main hydrophobic amino acid. The selection of tryptophan and biphenylalanine as bulky and lipophilic residues in the peptides was made to ensure a wide distribution of antimicrobial activity. All peptides adhere to the minimum motif for antimicrobial activity, and the increased bulkiness and lipophilicity of biphenylalanine over tryptophan ensures a higher antimicrobial efficacy of the Series 2 peptides compared to the Series 1 counterparts [34,35,36,37]. All peptides had a phenylalanine residue close to the amidated C-terminus. One peptide from each series was cyclized to investigate whether the anti-colonization efficacy was affected by the decrease in conformational freedom resulting from cyclization. Half of the peptides, **1c**, **1d**, **2c**, and **2d**, contained an azido-lysine residue used for a direct surface connection. The effect of linking the peptide directly to the Au–SAM or through an additional PEG linker was also included in the study using PEG200-linked peptides (**1a** and **2a**) and PEG400-linked peptides (**1b** and **2b**). An overview of the peptides prepared for the study is shown in Figure 3.

### 2.3. Intrinsic Antimicrobial Activity of the Peptides

The eight azidopeptide analogs designed for this study were screened against the Gram-positive bacteria *S. aureus* and *S. epidermidis* and the Gram-negative *E. coli* and *P. aeruginosa* reference strains to determine the intrinsic antimicrobial activity of the peptides as the minimum inhibitory concentration (MIC) (Table 1). The bacterial strains represent typical pathogens found in medical device-related biofilms.

The eight peptides displayed a substantial spread in MIC values, with the Gram-positive bacteria being more susceptible than the Gram-negatives. Some peptides, like **1c** and **2c**, displayed similar antimicrobial efficacy against all four bacteria in the panel, whereas other peptides (e.g., **1d**, **2a**, and **2d**) were quite selective against Gram-positive bacteria. The PEGylated tryptophan peptides **1a** (PEG200) and **1b** (PEG400) were modestly active against Gram-positive and inactive against Gram-negative bacteria. The linear peptide, **1c**, despite being modestly active against Gram-positive (MIC: 16–32 µg/mL) and Gram-negative (MIC: 64 µg/mL) bacteria, was more active than **1a** and **1b,** indicating that PEGylation slightly decreased the activity. The cyclization of **1c** to give **1d** improved the antimicrobial activity to 8 µg/mL against Gram-positive bacteria but remained the same as **1c** against *E. coli* (MIC: 64 µg/mL) and gave 128 µg/mL against *P. aeruginosa*.

Replacing the tryptophan residues in Series 1 with biphenylalanine residues increased the activity, as expected [36]. Peptide **2a** showed good activity against Gram-positive (MIC: 8 µg/mL) but was less active against the Gram-negative (MIC: 64 µg/mL) bacteria. Substituting PEG200 with PEG400 in peptide **2b** diminished activity similarly as in Series 1. Removing the PEG chain increased activity, as observed for **2c**. The cyclization of **2c** to give **2d** improved the activity against Gram-positive bacteria but became less active against Gram-negative bacteria. Peptide **2d** had potent activity against *S. aureus* (MIC: 4 µg/mL) and *S. epidermidis* (MIC: 2 µg/mL), but less activity against *E. coli* (MIC: 64 µg/mL) and was not active against *P. aeruginosa* (MIC: 256 µg/mL).

### 2.4. Characterization of Peptide Surfaces

The preparation of peptide-functionalized Au–SAM surfaces took place in two steps—self-assembly of the acetylene-terminated PEG monolater and the CuAAC functionalization with the antimicrobial peptides (Figure 2). To be able to reliably interpret the biological efficacy data, it is important to assess the integrity (the presence of the correct peptide) and the homogeneity of the surface. In the present study, two surface characterization methods were used—the measurement of the contact angle and spatially resolved ToF-SIMS mass spectrometry (ToF-SIMS imaging).

#### 2.4.1. Contact Angle

The contact angle is a measurement of the wettability of a surface, and as peptide functionalization of the Au–SAM surface is expected to alter the hydrophobicity of the surface, the contact angle is, hence, expected to be altered. A larger contact angle indicates a more hydrophobic surface (low surface energy), and the opposite indicates a hydrophilic surface (high surface energy) (Figure S25) [38]. To assess the homogeneity of the surface, the contact angle was determined for five droplets tested per peptide surface to get an average value and a standard deviation. The measured contact angles for the functionalized surfaces are shown in Table 2.

The observed contact angles did not vary significantly between the five droplets applied on each surface specimen, indicating that the surface was homogenous on the scale of the specimens. The contact angle, θ, of the control surface—the Au–SAM PEG-alkyne surface with no peptide functionalization—was 39.6°. The surfaces functionalized with the AMPs **1a**–**d** and **2a**–**d** showed a considerably higher θ than the control surface, verifying a significant increase in surface lipophilicity. This lipophilicity change is compatible with the AMPs being covalently linked to the surface by the CuAAC reaction. There were small differences between the peptides themselves with the same hydrophobic amino acids, but the biphenylalanine peptides (**2a**–**d**) showed an overall higher contact angle than the tryptophan peptides (**1a**–**d**) due to biphenylalanine being more hydrophobic than tryptophan [39].

#### 2.4.2. Surface Characterization by Spatially Resolved ToF-SIMS Mass Spectrometry

Spatially resolved time-of-flight secondary ion mass spectrometry (ToF-SIMS imaging) is a powerful technique for the characterization of surfaces that are modified by organic molecules in nanoscale layers, and it has successfully been used to verify surface attachment [40]. In this technique, a specimen is introduced into the ion source of a ToF-mass spectrometer, and the SIMS ion beam is scanned over the surface, providing spatially resolved SIMS mass spectra of the surface. In this manner, both the chemical composition and the homogeneity of the surface can be verified.

**Figure 4:**
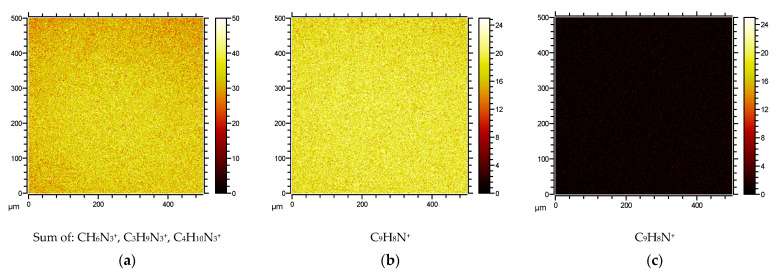
Typical ToF-SIMS images of an Au–SAM surface coated with azidopeptide-containing tryptophan, **1a**: (**a**) ion intensities for CH_6_N_3_^+^, C_3_H_9_N_3_^+^, and C_4_H_10_N_3_^+^ of arginine; (**b**) ion intensity of tryptophan indole-ion C_9_H_8_N^+^; (**c**) ion intensity image of control Au–SAM surface observed at the tryptophan-specific ion C_9_H_8_N^+^.

For the Trp-containing peptides **1a**–**d**, the SIMS mass spectra of the corresponding surfaces revealed the presence of signals due to the arginine and tryptophan residues. Arginine residues gave rise to ions originating from the side chain with CH_6_N_3_^+^ (the guanidinium group), as well as two additional ions, C_3_H_9_N_3_^+^ and C_4_H_10_N_3_^+^, including the carbon atoms of the arginine side chain. The tryptophan residues provided the side chain methyleneindole ion C_9_H_8_N^+^. The ToF-SIMS images of the peptide-modified surfaces were compared to the control Au–SAM surfaces, which had only the thiol-PEG-alkyne linked on the surface. As an example, Figure 4a shows the ToF-SIMS image of the Au–SAM surface functionalized with peptide **1a** when observing the arginine-specific ions. Figure 4b shows the image of the same surface when observed the tryptophan-specific ions. Figure 4c, on the other hand, shows the ToF-SIMS image when imaging the naïve (no peptide) Au–SAM surface by searching for the tryptophan-specific C_9_H_8_N^+^ ion. Arginine side chains and the indole group of tryptophan were observed on all the peptide surfaces, displaying high-intensity images by ToF-SIMS. Typical arginine and tryptophan surface data of peptide **1a** with the control group are displayed in Figure 4. Surface ToF-SIMS images for peptides **1b**–**d** are found in the Supplementary Material, Figure S26.

**Figure 5:**
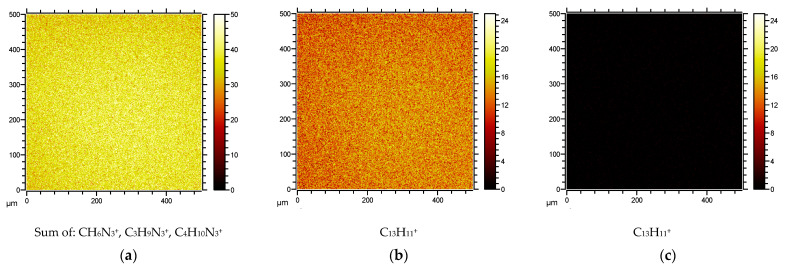
Typical ToF-SIMS images of an Au–SAM surface coated with azidopeptide-containing biphenylalanine, **2a**: (**a**) ion intensities for CH_6_N_3_^+^, C_3_H_9_N_3_^+^, and C_4_H_10_N_3_^+^ of arginine; (**b**) ion intensity of biphenylalanine-ion C_13_H_11_^+^; (**c**) ion intensity image of control Au–SAM surface observed at the biphenylalanine-specific ion C_13_H_11_^+^.

For biphanylalanine peptides **2a**–**d** linked to the Au–SAM surface, arginine and biphenylalanine moieties on the surface were located through ToF-SIMS imaging. The biphenylalanine residue was identified by the C_13_H_11_^+^ ion, which forms with a lower ionization efficiency than the arginine ions (Figure 5a,b). Figure 5c shows the control Au–SAM surface observed through the biphenylalanine ion, confirming the absence of a biphenylalanine peptide. Surface ToF-SIMS images for peptides **2b**–**d** are found in the Supplementary Material, Figure S27.

### 2.5. Anti-Colonization Efficacy of Peptide Modified Gold Surfaces

#### Certika

The antibacterial effectiveness of active surfaces can be tested with the proliferation assay Certika [41]. In short, this method consisted of the samples being washed in phosphate-buffered saline (PBS) in a 24-well plate before the test strains were added to each sample and incubated at 37 °C for 1 h to allow bacterial cells to adhere to the sample surface. Loosely bound bacteria were subsequently removed by washing in PBS before the samples were incubated in a minimum medium. After removal of the test samples, each well was supplemented with TSB complete medium. The bacterial growth (of the daughter cells) at 37 °C was recorded every 30 min for a period of 48 h by optical density (OD) measurements in a microtiter plate reader at a wavelength of 578 nm. Thus, the Certika method measured the anti-colonization efficacy of the surface as a prolonged onset time of bacterial growth. The antimicrobial effectiveness of an antimicrobial-coated surface is measured as the difference in the time required to reach the onset OD value of the active surface and the time needed to reach the onset OD value for the control surface, and hence, a prolonged time difference indicates an active surface. In the present study, the non-peptide Au–SAM surface was used as the inactive control, the onset OD value was set to 0.2, and the test bacterium, *S. epidermidis*, was assumed to divide once every 30 min. As an example, a time difference of 5 h in the net onset OD (in comparison to a blank sample) can be translated into the fact that it takes ten duplications/divisions (two duplications per hour) before the bacteria present on the active surface reaches the number of bacteria on the control surface. In 10 duplications, a single bacterium will give rise to 2^10^ bacteria, and hence, a time difference of 5 h equates to a reduction of 2^10^:1 (=1024:1) and/or ≈0.1% of the formed daughter cells on the active surface compared to the control.

**Figure 6:**
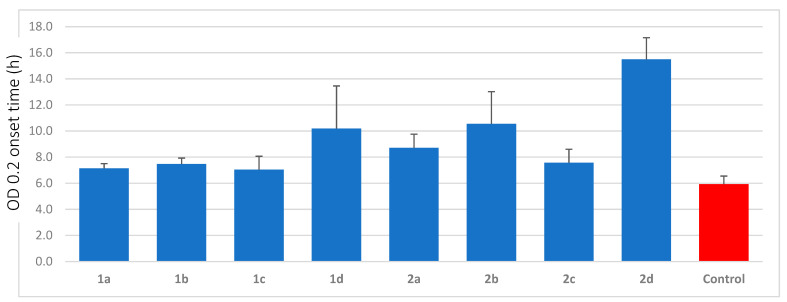
Certika assay with Au–SAM surfaces functionalized with peptides **1a**–**d** and **2a**–**d** as well as the control using the biofilm-forming bacterium *S. epidermidis* RP62A as a challenge. The diagram shows the OD 0.2 onset time in hours for all surfaces.

The Au–SAM surfaces functionalized with peptides **1a**–**d** and **2a**–**d** were tested against *S. epidermidis* RP62A using the Certika assay to determine their anti-colonization effect on the surfaces (Table S2). These peptide surfaces were compared to the non-peptide Au–SAM surface as the control group, shown in Figure 6.

Series 1 surfaces showed lower anti-colonization efficacy against *S. epidermidis* compared to the corresponding peptides belonging to Series 2. The best anti-colonization effect of the tryptophan series was observed for the cyclic peptide **1d**, with a prolonged onset time of 4 h, corresponding to 2^8^:1, that is, a 256:1 reduction of colonization. There was no difference between **1a** (PEG200) and **1c** (no PEG), while there was a slight increase in the onset time compared to that for **1b** (PEG400).

The anti-colonization efficacy pattern observed in the tryptophan peptides was also observed for the corresponding biphenylalanine peptides, although the anti-colonization efficacy was much higher. The cyclic biphenylalanine peptide **2d** displayed the highest activity of the entire panel of peptide-functionalized surfaces with a delayed net onset time of 10 h. Under the assumption of a generation time of 30 min, this delay corresponds to a reduction in the colonizing ability of 2^20^:1, or 1,048,576:1 (6 log scales). The PEG-length on the peptides also showed a correlation with enhanced activity, with PEG400 (**2b**) having the highest activity, followed by PEG200 (**2a**) and then non-PEGylated linear peptide **2c**.

## 3. Discussion

### 3.1. The Intrinsic Antimicrobial Activity of the Peptide Library

The antimicrobial peptides used in this study were designed based on the pharmacophore for short cationic antimicrobial peptides [27] to span a wide efficacy range from weak to very active peptides. Furthermore, the effect of cyclization of a linear peptide and the aspect of linking the peptides to the surface through an additional PEG tether or linking the peptides directly to the molecular monolayer were also investigated.

The pharmacophore predicts that all peptides prepared with at least two cationic charges and three bulky and lipophilic residues should have a minimum of antibacterial activity which is also observed in the present library. In some of the peptides, the tryptophan residues were substituted with biphenylalanine, with a subsequent change in bulk, geometry, and lipophilicity that favors enhanced antibacterial efficacy [34,35], an effect also observed in the present library, where the peptides in Series 2 were more effective than their Series 1 analogs. The effect of cyclization represents a loss of the N-terminal charge in the linear peptide as well as a substantial restriction on the conformational freedom of the peptide backbone, effects that turned out to be positive for the Gram-positive efficacy, but negative, activity-wise, against the Gram-negative bacteria. A general increase in antimicrobial activity upon cyclization has previously been reported for a small set of bacteria, however, the increase was the largest for *E. coli* [42], which is the opposite of what we observed. The effect of PEGylation upon the intrinsic antimicrobial activity was marginal when considering the mass increase connected with PEGylation. Overall, the library designed and prepared for the study represented a variety in antimicrobial efficacy from the very active peptides **1d**, **2c**, and **2d** to almost inactive peptides **1a**, **1b**, **2a**, and **2b**, as well as including structural diversity in the peptide library.

### 3.2. Surface Attachment of the Peptides

#### 3.2.1. Contact Angle and Surface Lipophilicity

The Cu(I)-catalyzed [3 + 2] cycloaddition (CuAAC) between the acetylenic terminus of the Au–SAM monolayer and the azide functionality of the peptides created a covalent, non-leaching peptide surface. The self-assembled monolayer is PEG-based; hence, the Au–SAM surface is hydrophilic in nature. The hydrophilicity of the SAM surface is evident in the low measured contact angle of 39.6°. The contact angle increased significantly to 49–55° upon the covalent attachment of the antimicrobial peptides. This increase in contact angle is a measurement of the increase in lipophilicity of the surface caused by attaching the amphipathic peptides to the surface. While the increase in contact angle of the surface upon peptide functionalization is substantial, the variation within the peptide series is smaller; the Series 1 tryptophan peptides varied between 49.1° and 52.2°, whereas the Series 2 biphenylalanine peptides were more lipophilic, with contact angles between 53.5° and 54.9°. Although the Series 2 peptides were more efficacious against the bacteria than those in Series 1—fitting the general picture that lipophilic peptides are more active than their less lipophilic counterpart—there is seemingly no correlation between the contact angle and the peptide activity within each group.

#### 3.2.2. Verification of Surface Integrity and Homogeneity by Spatially Resolved ToF-SIMS Mass Spectrometry

The interpretation of the anti-colonization efficacy of the various peptide-modified surfaces is highly dependent on verifying the integrity—whether the peptide is present on the surface—and the homogeneity of the surface—that the peptides coupled to the surface are evenly spread. ToF-SIMS imaging is a premier method for such an analysis [40]. The technique provides a mass resolved 2D-map (image) of the surface specimen. When the masses selected for the imaging are among the characteristic ions for each amino acid in the sequence, the combined maps provide the spatial distribution of the peptides, as shown in Figure 4 and Figure 5. The data unequivocally shows the presence of the specific peptides on the surface, and the surfaces are homogenous in nature. A drawback with the method is that it is not quantitative, making comparisons between specimens difficult [43]. However, the homogeneity within a sample is a good indication that we can consider that there are no gross differences in the surface density of the peptides between the different surfaces, but that the absolute surface density is an unknown factor. On this basis, that is, that the peptides are connected to the Au–SAM surfaces in a manner that is similar for all peptides, we could start interpreting the anti-colonization activity of the different surfaces.

### 3.3. Anti-Colonization Efficacy

The Certika test that was selected to assay the anti-colonization efficacy of the peptide-modified surfaces is a rigorous quantitative test that is based on measuring the quantitative regrowth of surface adherent bacteria after a bacterial challenge. The Certika method is particularly valuable as it can measure a wide variation of anti-colonization efficacy without the dilution of the assay material.

The Certika test was applied to all eight surfaces using *S. epidermidis* as the challenge organism. *S. epidermidis* was selected because it is a bacterium with a large potential for surface colonization and subsequent biofilm formation [44]. The Certika test confirmed that the anti-colonization of all surfaces increased after peptide linkage, but the efficacy varied to a large degree. The trends in the anti-colonization efficacy of the peptide-functionalized surfaces grossly followed the intrinsic MIC values for the individual peptides. The major break in the correlation was that the PEG400 peptides became more efficacious than the shorter PEG200 peptides, a result suggesting that the increased motional freedom of the peptides with a longer tether to the surface was beneficial for the anti-colonization efficacy. The trend was even extended to the **1c** and **2c** peptides, where the peptides were connected directly to the SAM surface without the use of an additional PEG tether, although a direct comparison is more difficult, as the **1c** and **2c** peptides were connected close to the C-terminus and had an additional charge compared to the peptides **1a**, **2a**, **1b**, and **2b**, which were linked to the surface through a PEG unit connected to the N-terminus.

The most surprising result, however, was the large difference observed in the anti-colonization efficacy compared to the relatively narrow span of the intrinsic antimicrobial efficacy of the non-coupled peptides. While the antibacterial efficacy of the eight peptides in the library covered a range of 2–128 µg/mL, the anti-colonization efficacy varied from 2^2^:1 for surfaces with **1a** and **1c** to 2^10^:1 for a surface prepared with **2d**, or, in log sales, a variation between 0.6 log and 6 log.

An anti-colonization efficacy in the order of 6 log units is well within what would be needed for practical utilization of covalently anchored peptides to create an anti-colonization surface. Admittedly, the surface used here was a model surface, and more work is needed to translate these promising results into a practical and general method for peptide functionalization of surfaces. Furthermore, the analytical techniques used in the present study do not provide a quantification of the surface density of the peptides. On the other hand, the AMP-functionalized Au–SAM method provides a surface with high homogeneity and chemical integrity, allowing for the determination of the influence of the intrinsic antimicrobial efficacy of the attached peptides and the anti-colonization efficacy they provide on a surface. The results, so far, have revealed a surprising effect where modest differences in antimicrobial efficacy translated into large changes in anti-colonization activity.

## 4. Materials and Methods

### 4.1. Materials

Rink amide HL resin, 2-chlorotrityl chloride resin, and natural Fmoc-protected amino acids were bought from Novabiochem. Fmoc-Bip-OH was purchased from Iris Biotech. Other chemicals used in standard Fmoc solid phase peptide synthesis (SPPS) were bought from Sigma-Aldrich. The starting material, reactants, and solvents for the synthesis of the PEG linker were purchased from Sigma-Aldrich. Pre-diced gold (Au)-coated Si-wafers (10 × 10 × 0.5 mm) were purchased from ConScience AB, Gothenburg, Sweden. The Au surface was protected by polymer S1813 during shipment and handling. α-Thio-PEG600-ω-alkyne was purchased from Nanocs, NY, USA, while the rest of the chemicals for CuAAC were bought from Sigma-Aldrich.

### 4.2. Experimental Method

#### 4.2.1. Synthesis of Azide and Carboxylic Acid Terminal-Conjugated Polyethylene Glycol

**Figure 7:**
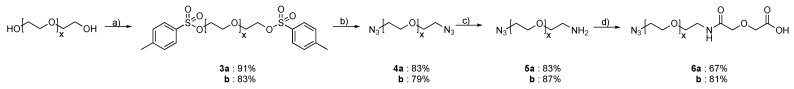
Synthesis of a modified PEG (average Mw: 200 (Series **a**) and 400 (Series **b**)) moiety with azido and carboxylic acid terminals: (**a**). TsCl and. KOH in DCM at rt overnight; (**b**) NaN_3_ in Et_2_O at 80 °C overnight; (**c**) PPh_3_ in Et_2_O:1M HCl (1:1) rt overnight; (**d**) 2.0 eq. diglycolic anhydride and 0.2 eq. DMAP in DCM rt overnight. The chemical yield is given under each compound.

The preparation of the α-carboxyl-PEG-ω-alkynes **6a** and **b** were prepared from PEG200 and PEG400, respectively, through a four-step sequence (a–d) outlined in Figure 7.

***O*,*O′*-Bis(tosyloxy)polyethylene glycols**

Polyethylene glycol (PEG, average Mw 200) (0.5 g, 0.0025 mol) was dissolved in 50 mL dichloromethane (DCM) and cooled on an ice bath for 15 min. Powdered KOH (8.0 eq., 1.1221 g, 0.02 mol) was added slowly before adding 4-toluenesulfonyl chloride (3.0 eq., 1.4298 g, 0.0075 mol) to the ice-cold solution. The reaction was carried out overnight at room temperature before quenching with 15 mL of ice-cold water. The reaction mixture was extracted with 10 mL of DCM three times, dried over MgSO_4_, filtered, and concentrated under vacuum. The crude product was purified by silica column chromatography (MeOH/DCM, 1:10) to give **3a** (1.157 g, 91%) as a transparent colorless oil. ^1^H NMR (400 MHz, CDCl_3_): δ 7.83 (dd, *J* = 8.3, 1.4 Hz, 4H), 7.37 (d, *J* = 8.0 Hz, 4H), 4.20–4.15 (m, 4H), 3.74–3.54 (m, 14H), 2.48 (s, 6H) (Figure S1). HRMS (ESI): calculated for C_22_H_30_O_9_S_2_Na^+^ [M + Na]^+^ 525.1229; found 525.1226.

Synthesis of PEG average Mw 400 (1.0 g, 0.0025 mol) was synthesized using the same method as above to give **3b** (1.468 g, 83%) as a transparent colorless oil. ^1^H NMR (400 MHz, CDCl_3_): δ 7.82–7.75 (m, 4H), 7.33 (d, *J* = 8.1 Hz, 4H), 4.14 (dd, *J* = 5.7, 4.0 Hz, 4H), 3.67 (dd, *J* = 5.6, 4.1 Hz, 4H), 3.65–3.58 (m, 17H), 3.57 (s, 8H), 2.44 (s, 6H) (Figure S2). HRMS (ESI): calculated for C34H_54_O_15_S_2_Na^+^ [M + Na]^+^ 789.2802; found 789.2796

***O*,*O′*-Bis(2-azidoethyl)polyethylene glycols**

Compound **3a** (15.957 g, 0.031 mol) was dissolved in 80 mL dimethylformamide (DMF). To the stirred solution, sodium azide (3.0 eq., 6.125 g, 0.093 mol) was added slowly and the mixture refluxed at 80 °C overnight. Excess sodium azide was quenched with 100 mL of ice-cold water. The product was extracted with diethyl ether, dried over MgSO_4_, filtered, and concentrated under vacuum to give crude **4a** (6.535 g, 83%) as a transparent colorless oil. The product was used without further purification. ^1^H NMR (400 MHz, CDCl_3_): δ 3.71–3.64 (m, 14H), 3.39 (td, *J* = 5.1, 2.4 Hz, 4H) (Figure S3). HRMS (ESI): calculated for C_14_H_28_N_6_O_6_Na^+^ [M + Na]^+^ 399.1968; found 399.1954

The same method was applied for the preparation of **4b** from **3b** (4.843 g, 0.0068 mol) to give **4b** (2.441 g, 79%) as a transparent colorless oil. ^1^H NMR (400 MHz, CDCl_3_) δ 3.69–3.63 (m, 30H), 3.38 (t, *J* = 5.1 Hz, 4H) (Figure S4). HRMS (ESI): calculated for C_24_H_48_N_6_O_11_K^+^ [M + K]^+^ 635.3018; found 635.3006

***O*-(2-Aminoethyl)-*O′*-(2-azidoethyl)polyethylene glycols**

To a stirred solution of **4a** (5.537 g, 0.022 mol) in 50 mL diethyl ether, 1M HCl (50 mL) and triphenylphosphine (1.0 eq., 5.804 g, 0.022 mol) were added and the resulting mixture was stirred overnight at room temperature. White solids of triphenylphosphine oxide were removed by filtration, and the filtrate was extracted with diethyl ether to remove residues of triphenylphosphine oxide. The pH of the aqueous phase was adjusted by KOH until pH ~12, extracted with 15 mL DCM, dried over MgSO_4_, filtered, and concentrated under vacuum to give crude **5a** (4.157 g, 83%) as a transparent colorless oil. ^1^H NMR (400 MHz, CDCl_3_): δ 3.72–3.61 (m, 14H), 3.52 (ddd, *J* = 8.7, 5.7, 4.2 Hz, 2H), 3.40 (dt, *J* = 8.4, 4.9 Hz, 2H), 2.88 (q, *J* = 5.3 Hz, 2H), 1.91 (s, 2H) (Figure S5). HRMS (ESI): calculated for C_14_H_31_N_4_O_6_^+^ [M + H]^+^ 351.2238; found 351.2245.

The same method was applied for the preparation of **5b** from **4b** (2.441 g, 0.0054 mol). Compound **5b** (1.999 g, 87%) was obtained as a yellow oil. ^1^H NMR (400 MHz, CDCl_3_): δ 3.61–3.46 (m, 28H), 3.39 (t, *J* = 5.2 Hz, 2H), 3.26 (t, *J* = 5.0 Hz, 2H), 2.73 (t, *J* = 5.2 Hz, 2H), 2.08 (s, 3H) (Figure S6). HRMS (ESI): calculated for C_24_H_51_N_4_O_11_^+^ [M + H]^+^ 571.3549; found 571.3532

***O*-(2-Azidoethyl)-*O*-[2-(diglycolyl-amino)ethyl]polyethylene glycols**

To a stirred solution of **5a** (3.516 g, 0.016 mol) in 35 mL DCM, diglycolic anhydride (2.0 eq., 3.643 g, 0.032 mol) and 4-dimethylaminopyridine (0.2 eq., 0.352 g, 0.0032 mol) were added and the reaction mixture was stirred for 4 h. Ethylenediamine (2.0 eq., 1.886 g, 0.032 mol) was added and stirring was continued overnight to remove the excess diglycolic anhydride. DCM (30 mL) was added before washing the organic phase with 15 mL of 1M HCl three times. The combined acidic aqueous phase was extracted with 5x15 mL dichloromethane. The organic layer was dried over MgSO_4,_ filtered and concentrated under vacuum to give **6a** (3.615 g, 67%) as a pink oil. ^1^H NMR (400 MHz, CDCl_3_): δ 10.15 (s, 1H), 4.08 (s, 2H), 4.05 (s, 2H), 3.65–3.51 (m, 14H), 3.49 (t, *J* = 5.4 Hz, 2H), 3.41 (q, *J* = 5.3 Hz, 2H), 3.30 (t, *J* = 4.9 Hz, 2H) (Figure S7). HRMS (ESI): calculated for C_16_H_30_N_4_O_9_Na^+^ [M + Na]^+^ 445.1910; found 445.1925.

The same method was applied for the preparation of **6b** from **5b** (1.979 g, 0.005 mol). Compound **6b** (2.060 g, 81%) was obtained as a pink oil. ^1^H NMR (400 MHz, CDCl_3_): δ 7.66 (s, 1H), 4.16 (s, 2H), 4.11 (s, 2H), 3.71–3.60 (m, 33H), 3.58–3.54 (m, 2H), 3.51 (q, *J* = 5.1 Hz, 2H), 3.38 (t, *J* = 5.1 Hz, 2H) (Figure S8). HRMS (ESI): calculated for C_22_H_42_N_4_O_12_Na^+^ [M + Na]^+^ 577.2697; found 577.2689.

#### 4.2.2. Synthesis of Linear Azidopeptides and Azido PEG Peptides

The linear azidopeptides were assembled on a Rink Amide HL 100–200 mesh (loading: 0.98 mmol/g) based on an Fmoc solid-phase peptide synthesis technique. The scale varied from 0.18 mmol to 0.21 mmol.

Loading of Rink Amide resin and coupling: Rink Amide HL (0.98 mmol/g) was swelled at 70 °C for 20 min. A solution of 20% piperidine in DMF was added to the resin to remove the Fmoc group from the resin. Fmoc amino acids (4.00 eq.) were coupled with *O*-(benzotriazol-1-yl)-*N,N*,*N*′,*N*′-tetramethyluronium hexafluorophosphate (HBTU, 3.92 eq.), 1-hydroxybenzotriazole hydrate (HOBt, 4.00 eq.), and *N,N*-diisopropylethylamine (DIPEA, 8.0 eq.). The removal of N-terminal Fmoc was carried out with piperidine (20%) in DMF before coupling the next amino acid. Fmoc-Phe-OH (4.0 eq.) and Fmoc-Trp(Boc)-OH (4.0 eq.) were coupled at 70 °C for 5 min and Fmoc-Bip-OH (4.0 eq.) was coupled at 70 °C for 15 min. Fmoc-Arg(Pbf)-OH (4.0 eq.) was coupled at room temperature for 60 min and Fmoc-Lys(N_3_) was coupled at room temperature for 16 h. N_3_-PEG200-COOH (4.0 eq.) or N_3_-PEG400-COOH (4.0 eq.) were coupled with HBTU (3.92 eq.) and HOBt (4.0 eq.) at room temperature for 16 h. After the last coupling, non-PEGylated peptides were removed from the Fmoc group, and the resin was washed with MeOH and DCM before being placed in a desiccator to dry overnight.

Cleavage from the resin and side-chain deprotection: A solution of TFA (trifluoroacetic acid)/TIS (triisopropylsilane)/H_2_O (95:2.5:2.5, 10 mL) was added to the resin twice to remove the protecting groups. The combined solutions were evaporated under reduced pressure before adding ice-cold diethyl ether to precipitate the peptide. The crude peptides were washed 3 times with diethyl ether before purifying the peptides by reversed-phase high-performance liquid chromatography (RP-HPLC) to achieve a purity of ≥95%.

Following the procedure in Section 4.2.2 at a 0.21 mmol scale, **1a** was isolated as a white powder (44.4 mg, 17.2%) (Figure S17). ^1^H NMR (400 MHz, DMSO-*d*_6_) δ 10.80 (d, *J* = 2.3 Hz, 2H), 8.13 (dd, *J* = 9.9, 7.5 Hz, 2H), 8.05 (td, *J* = 7.2, 6.0, 3.7 Hz, 2H), 7.98 (d, *J* = 8.2 Hz, 1H), 7.91 (d, *J* = 8.1 Hz, 1H), 7.61 (q, *J* = 5.3 Hz, 2H), 7.57 (d, *J* = 7.9 Hz, 2H), 7.45–7.41 (m, 1H), 7.32 (dd, *J* = 8.0, 6.3 Hz, 3H), 7.25–7.19 (m, 6H), 7.15 (dt, *J* = 5.7, 3.2 Hz, 5H), 7.09 (d, *J* = 2.4 Hz, 2H), 7.08–7.02 (m, 4H), 6.95 (t, *J* = 7.4 Hz, 3H), 4.55 (dq, *J* = 8.4, 4.7, 3.9 Hz, 2H), 4.47 (td, *J* = 8.4, 5.2 Hz, 2H), 4.34–4.20 (m, 7H), 3.96 (s, 4H), 3.61–3.55 (m, 2H), 3.55–3.48 (m, 8H), 3.43 (td, *J* = 6.0, 4.4 Hz, 2H), 3.40–3.35 (m, 2H), 3.30–3.23 (m, 2H), 3.13 (dd, *J* = 15.2, 4.4 Hz, 2H), 3.03 (dt, *J* = 19.7, 7.7 Hz, 8H), 2.96–2.79 (m, 3H), 1.62 (ddd, *J* = 13.7, 9.5, 6.3 Hz, 2H), 1.55–1.32 (m, 8H) (Figure S9). HRMS (ESI): calculated for C_57_H_82_N_18_O_12_^2+^ [M + 2H]^2+^ 605.3175; found 605.3186.

Following the procedure in Section 4.2.2 at a 0.21 mmol scale, **1b** was isolated as a white powder (31.5 mg, 18.3%) (Figure S18). ^1^H NMR (600 MHz, DMSO-*d*_6_): δ 10.79 (d, *J* = 2.4 Hz, 2H), 8.14 (d, *J* = 7.4 Hz, 1H), 8.11 (d, *J* = 7.5 Hz, 1H), 8.07–8.03 (m, 2H), 7.98 (d, *J* = 8.1 Hz, 1H), 7.91 (d, *J* = 8.1 Hz, 1H), 7.57 (dd, *J* = 11.2, 7.0 Hz, 4H), 7.44–7.42 (m, 1H), 7.32 (t, *J* = 8.7 Hz, 3H), 7.23 (q, *J* = 5.9, 4.7 Hz, 6H), 7.15 (ddd, *J* = 13.9, 6.1, 2.1 Hz, 4H), 7.09 (d, *J* = 2.4 Hz, 1H), 7.05 (td, *J* = 7.3, 4.0 Hz, 3H), 6.95 (t, *J* = 7.4 Hz, 3H), 4.55 (tt, *J* = 8.4, 3.8 Hz, 2H), 4.47 (td, *J* = 8.4, 5.2 Hz, 1H), 4.31 (td, *J* = 8.4, 5.4 Hz, 1H), 4.23 (q, *J* = 7.2 Hz, 1H), 3.97–3.94 (m, 4H), 3.59 (q, *J* = 3.9, 2.9 Hz, 2H), 3.57–3.46 (m, 33H), 3.42 (t, *J* = 6.0 Hz, 3H), 3.38 (t, *J* = 5.0 Hz, 3H), 3.26 (q, *J* = 6.0 Hz, 3H), 3.15–3.10 (m, 2H), 3.02 (tq, *J* = 19.9, 6.1 Hz, 8H), 2.91 (dd, *J* = 15.0, 9.4 Hz, 1H), 2.83 (dd, *J* = 13.9, 8.7 Hz, 1H), 1.66–1.58 (m, 2H), 1.49 (tdd, *J* = 13.7, 9.9, 5.9 Hz, 3H), 1.38 (tdd, *J* = 15.6, 11.6, 6.7 Hz, 5H) (Figure S10). HRMS (ESI): calculated for C_69_H_106_N_18_O_18_^2+^ [M + 2H]^2+^ 737.3961; found 737.3957.

Following the procedure in Section 4.2.2 at a 0.18 mmol scale, **1c** was isolated as a white powder (18.0 mg, 9.9%) (Figure S19). ^1^H NMR (400 MHz, DMSO-*d*_6_) δ 10.85 (d, *J* = 2.4 Hz, 1H), 10.78 (d, *J* = 2.4 Hz, 1H), 8.54 (d, *J* = 7.7 Hz, 1H), 8.35 (d, *J* = 7.7 Hz, 1H), 8.14–8.01 (m, 6H), 7.71 (t, *J* = 5.9 Hz, 1H), 7.66 (d, *J* = 7.8 Hz, 1H), 7.63–7.55 (m, 2H), 7.33 (dd, *J* = 8.1, 2.7 Hz, 3H), 7.28–7.19 (m, 7H), 7.17–7.10 (m, 4H), 7.09–7.02 (m, 4H), 6.95 (dt, *J* = 12.8, 7.5 Hz, 3H), 4.69–4.60 (m, 1H), 4.57 (tt, *J* = 7.6, 3.7 Hz, 2H), 4.30 (q, *J* = 7.1 Hz, 1H), 4.18 (td, *J* = 8.2, 5.3 Hz, 1H), 3.73 (dt, *J* = 11.4, 4.8 Hz, 1H), 3.53–3.46 (m, 2H), 3.26 (t, *J* = 6.9 Hz, 2H), 3.18–3.10 (m, 1H), 3.11–2.99 (m, 6H), 2.94 (td, *J* = 15.3, 9.3 Hz, 2H), 2.83 (dd, *J* = 14.0, 8.8 Hz, 1H), 1.66 (tt, *J* = 13.7, 6.6 Hz, 4H), 1.47 (qd, *J* = 13.1, 10.7, 6.3 Hz, 8H), 1.29 (tt, *J* = 15.4, 6.5 Hz, 2H) (Figure S11). HRMS (ESI): calculated for C_49_H_67_N_18_O_6_^+^ [M + H]^+^ 1003.5485; found 1003.5492.

Following the procedure in Section 4.2.2 at a 0.21 mmol scale, **2a** was isolated as a white powder (33.6 mg, 12.8%) (Figure S21). ^1^H NMR (400 MHz, DMSO-*d*_6_): δ 8.28 (d, *J* = 7.8 Hz, 2H), 8.09–7.97 (m, 4H), 7.69 (t, *J* = 5.7 Hz, 1H), 7.61 (t, *J* = 8.2 Hz, 6H), 7.55 (d, *J* = 8.0 Hz, 3H), 7.50 (d, *J* = 7.9 Hz, 3H), 7.45 (qd, *J* = 7.9, 6.3, 1.9 Hz, 7H), 7.38–7.30 (m, 6H), 7.28 (d, *J* = 8.1 Hz, 3H), 7.23 (d, *J* = 4.5 Hz, 6H), 7.18–7.11 (m, 3H), 4.58 (dtd, *J* = 13.2, 8.4, 4.2 Hz, 2H), 4.50 (td, *J* = 8.2, 5.3 Hz, 1H), 4.30 (dq, *J* = 14.2, 7.8, 7.3 Hz, 2H), 3.96 (s, 2H), 3.94 (s, 2H), 3.60–3.54 (m, 3H), 3.50 (ddd, *J* = 14.7, 7.5, 4.0 Hz, 9H), 3.44–3.33 (m, 5H), 3.24 (q, *J* = 5.9 Hz, 2H), 3.12–2.97 (m, 8H), 2.84 (dp, *J* = 13.8, 9.3, 7.9 Hz, 3H), 1.71–1.59 (m, 2H), 1.59–1.31 (m, 7H) (Figure S13). HRMS (ESI): calculated for C_63_H_84_N_16_O_11_^2+^ [M + 2H]^2+^ 620.3247; found 620.3240.

Following the procedure in Section 4.2.2 at a 0.21 mmol scale, **2b** was isolated as a white powder (44.0 mg, 14.7%) (Figure S22). ^1^H NMR (600 MHz, DMSO-*d*_6_): δ 8.30–8.26 (m, 2H), 8.06 (d, *J* = 8.2 Hz, 1H), 8.05–8.02 (m, 2H), 7.99 (d, *J* = 8.1 Hz, 1H), 7.68 (t, *J* = 5.7 Hz, 1H), 7.64–7.59 (m, 5H), 7.55 (d, *J* = 8.0 Hz, 2H), 7.50 (d, *J* = 8.0 Hz, 2H), 7.44 (ddd, *J* = 15.6, 8.5, 2.5 Hz, 6H), 7.37–7.32 (m, 5H), 7.28 (d, *J* = 8.0 Hz, 3H), 7.23 (d, *J* = 5.2 Hz, 5H), 7.15 (td, *J* = 5.4, 3.0 Hz, 2H), 7.13 (t, *J* = 2.9 Hz, 1H), 4.58 (dtd, *J* = 19.2, 8.5, 4.5 Hz, 2H), 4.50 (td, *J* = 8.3, 5.3 Hz, 1H), 4.31 (ddt, *J* = 21.4, 14.3, 6.3 Hz, 2H), 3.96 (s, 2H), 3.94 (d, *J* = 1.7 Hz, 2H), 3.59 (dtd, *J* = 4.8, 3.5, 2.0 Hz, 3H), 3.57–3.45 (m, 30H), 3.41 (t, *J* = 6.0 Hz, 2H), 3.38 (t, *J* = 4.9 Hz, 2H), 3.27–3.22 (m, 2H), 3.07 (q, *J* = 6.7 Hz, 3H), 3.04 (d, *J* = 5.0 Hz, 2H), 3.01 (dt, *J* = 12.7, 5.8 Hz, 3H), 2.89–2.77 (m, 3H), 1.70–1.61 (m, 2H), 1.59–1.31 (m, 7H) (Figure S14). HRMS (ESI): calculated for C_73_H_104_N_16_O_16_^2+^ [M + 2H]^2+^ 730.3903; found 730.3900.

Following the procedure in Section 4.2.2 at a 0.18 mmol scale, **2c** was isolated as a white powder (98.0 mg, 50.5%) (Figure S23). ^1^H NMR (400 MHz, DMSO-*d*_6_) δ 8.63 (d, *J* = 7.9 Hz, 1H), 8.47 (d, *J* = 7.9 Hz, 1H), 8.28 (d, *J* = 7.8 Hz, 1H), 8.11 (d, *J* = 7.9 Hz, 3H), 8.05 (d, *J* = 8.1 Hz, 1H), 7.76 (t, *J* = 5.8 Hz, 1H), 7.71 (t, *J* = 5.6 Hz, 1H), 7.66–7.60 (m, 4H), 7.54 (dd, *J* = 8.3, 2.4 Hz, 4H), 7.45 (dd, *J* = 8.4, 6.9 Hz, 5H), 7.39–7.32 (m, 7H), 7.28 (d, *J* = 2.2 Hz, 2H), 7.26–7.18 (m, 6H), 7.16–7.10 (m, 2H), 7.10–7.05 (m, 1H), 4.70–4.56 (m, 3H), 4.31 (q, *J* = 7.2 Hz, 1H), 4.19 (td, *J* = 8.2, 5.3 Hz, 1H), 3.82–3.73 (m, 1H), 3.27 (t, *J* = 6.9 Hz, 2H), 3.05 (dq, *J* = 18.3, 7.6, 5.9 Hz, 7H), 2.91–2.76 (m, 3H), 1.66 (qd, *J* = 9.1, 8.6, 3.8 Hz, 4H), 1.59–1.39 (m, 8H), 1.38–1.23 (m, 2H) (Figure S15). HRMS (ESI): calculated for C_57_H_73_N_16_O_6_^+^ [M + H]^+^ 1077.5894; found 1077.5907.

#### 4.2.3. Synthesis of Cyclic Azidopeptides

The cyclic azidopeptides were prepared from the fully protected linear analogs assembled on 2-chlorotrityl chloride resin.

Activation and loading of 2-chlorotrityl chloride resin: 2-chlorotrityl chloride resin (1.63 mmol/g, 0.18 mmol scale) was swelled for an hour in DCM. A solution of thionyl chloride (1.2 eq.) was added to the resin under argon and stirred slowly for 2 h before washing the resin thoroughly with DCM. Fmoc-Phe-OH (3.00 eq.) was coupled to the resin with DIPEA (6.0 eq.) in DCM and stirred slowly overnight at room temperature.

Capping, removal of Fmoc, and amino acid coupling: The remaining uncoupled sites were capped with DCM/MeOH/DIPEA (80:15:5, 10 mL) for 30 min. The Fmoc group was removed using a solution of 20% piperidine in DMF. The amino acids (4.0 eq.) were coupled with HBTU (3.92 eq), HOBt (4.0 eq.), and DIPEA (8.0 eq.) at 70 °C for 5 min (Fmoc-Bip-OH: 15 min) with the exception of Fmoc-Arg(Pbf)-OH and Fmoc-Lys(N_3_)-OH. Fmoc-Arg(Pbf)-OH and Fmoc-Lys(N_3_)-OH were coupled at room temperature for 1 h and 16 h, respectively. After coupling the final amino acid, the N-terminal Fmoc group was removed, and the resin was washed with MeOH and DCM before being left to dry overnight in a desiccator.

Cleavage from the resin and cyclization: Hexafluoroisopropanol (HFIP) in DCM (3:7, 5 mL) was added to the resin and stirred slowly for 45 min. The process was repeated two times. The combined solution was evaporated under vacuum. The resulting peptide was dissolved in 10 mL of DMF, and DIPEA (6.0 eq.) was added. Benzotriazole-1-yl-oxytris-pyrrolidino-phosphonium hexafluorophosphat (PyBOP, 3.0 eq.) was dissolved in 200 mL of DMF and stirred rapidly before adding the peptide dropwise. The reaction was monitored by MS until completion. The solvents were evaporated under reduced pressure.

Side-chain deprotection and diethyl ether precipitation: A solution of TFA/TIS/H_2_O (95:2.5:2.5, 10 mL) was added to the peptide and stirred for 3 h. The solvents were evaporated under reduced pressure before ice-cold diethyl ether was added. The precipitate was washed three times with diethyl ether and the crude peptide was purified by RP-HPLC.

Following the procedure in Section 4.2.3 at a 0.18 mmol scale, **1d** was isolated as a white powder (22 mg, 12.3%) (Figure S20). ^1^H NMR (400 MHz, DMSO-*d*_6_) δ 10.74 (d, *J* = 2.3 Hz, 1H), 10.69 (s, 1H), 8.11–7.96 (m, 4H), 7.90 (d, *J* = 7.7 Hz, 1H), 7.52 (d, *J* = 7.8 Hz, 1H), 7.48–7.38 (m, 3H), 7.35 (d, *J* = 5.8 Hz, 1H), 7.32–7.09 (m, 11H), 7.07 (d, *J* = 2.3 Hz, 2H), 7.05–6.95 (m, 4H), 6.91 (q, *J* = 7.2 Hz, 3H), 4.31–4.18 (m, 3H), 3.95 (s, 1H), 3.85–3.73 (m, 2H), 3.22 (td, *J* = 6.9, 1.9 Hz, 3H), 3.17–3.10 (m, 2H), 3.09–2.85 (m, 9H), 1.70–1.37 (m, 9H), 1.34–1.07 (m, 6H) (Figure S12).

Following the procedure in Section 4.2.3 at a 0.18 mmol scale, **2d** was isolated as a white powder (65 mg, 34.0%) (Figure S24). ^1^H NMR (400 MHz, DMSO-*d*_6_) δ 8.28 (d, *J* = 7.6 Hz, 1H), 8.20 (q, *J* = 7.5, 6.5 Hz, 4H), 8.13 (d, *J* = 7.1 Hz, 1H), 7.67–7.50 (m, 11H), 7.43 (td, *J* = 7.7, 3.1 Hz, 5H), 7.37–7.24 (m, 10H), 7.24–7.18 (m, 4H), 4.40–4.28 (m, 2H), 4.21 (q, *J* = 7.6 Hz, 1H), 4.06 (q, *J* = 7.1 Hz, 1H), 3.92 (q, *J* = 7.1 Hz, 2H), 3.28 (td, *J* = 6.8, 2.5 Hz, 2H), 3.25–3.17 (m, 2H), 3.13–2.93 (m, 8H), 1.89–1.44 (m, 9H), 1.27 (tdd, *J* = 27.7, 16.1, 7.1 Hz, 7H) (Figure S16). HRMS (ESI): calculated for C_57_H_70_N_18_O_5_^+^ [M + H]^+^ 1060.5628; found 1060.5637.

#### 4.2.4. HPLC

Peptides **1a**–**b** and **2a**–**b** were purified using RP-HPLC on a Supelco Ascentis C_18_ column (10 μm, 10 cm × 21.2 mm, flow rate 7 mL/min) and peptides **1c**–**d** and **2c**–**d** were purified using RP-HPLC on a YMC-Triart C_18_ column (5 µm, 20 × 150 mm, flow rate of 11 mL/min) with a mixture of water and acetonitrile (both containing 0.1% TFA) as eluent. The peptides were analyzed by RP-HPLC using a Supelco Ascentis Express C18 column (2.7 μm, 10 cm × 3.0 mm, flow rate of 1 mL/min) and positive ion electrospray mass spectrometry.

#### 4.2.5. Preparation of Au Surface and Copper(I)-Catalyzed Alkyne-Azide Cycloaddition

The Au surfaces were washed with MeOH (50 mL × 4), dried with N_2_, placed in a UV oven for 20 min, and then washed with Milli-Q^®^ H_2_O. A beaker with 25 mL Milli-Q^®^ H_2_O and 5 mL ammonia (33% solution) was heated to 75 °C before adding the Au surfaces. After 5 min, 5 mL of hydrogen peroxide was added before simmering for 12 min. Each surface was washed thoroughly with Milli-Q^®^ H_2_O before adding 3 mL of an ethanolic solution of HS-PEG-Alkyne (Mw600, 0.08 mg/mL) and left to react overnight at room temperature. The following day, each surface was washed thoroughly with EtOH (5 mL × 5), and Milli-Q^®^ H_2_O (5 mL × 5) and dried with N_2_. Solutions of the peptides (100 µM) in Dulbecco’s phosphate-buffered saline (DPBS), ascorbic acid (1 mM) in DPBS, and the copper (CuSO_4_, 150 µM) with tris-hydroxypropyltriazolylmethylamine (750 µM) and HCl (1 mM) in DPBS were prepared. To the tubes with the dried Au surfaces, 700 µL of peptide solution, 700 µL of copper solution, and 700 µL of ascorbic acid solution were added and left to react overnight at room temperature. The control surfaces received 700 µL of peptide solution and 1400 µL of DPBS.

Upon completion of the copper(I)-catalyzed alkyne-azide cycloaddition, each surface was washed thoroughly with Milli-Q^®^ H_2_O (25 mL × 5) and then dried with N_2_.

#### 4.2.6. Minimal Inhibitory Concentration Determinations

The minimal inhibitory concentration (MIC) of peptides **1a**, **1b**, **2a**, and **2b** were determined by broth microdilution according to the Clinical Laboratory Standard Institute (CLSI) method M07-A9. The MICs of the peptides **1c**, **1d**, **2c,** and **2d** were determined by broth dilution according to the European Committee for Antimicrobial Susceptibility Testing (EUCAST) [45]. Final concentrations of 0.5–256 µg/mL were tested against a panel of reference bacteria—*S. aureus* (ATCC 9144), *S. epidermidis* (1457), *E. coli* (ATCC 25922), and *P. aeruginosa* (ATCC 27853).

Each strain was tested in biological triplicate. The MIC values were defined as the lowest concentration of the peptides resulting in no visual growth.

#### 4.2.7. Certika Assay

The Certika assay was adopted from Bruenke et al. [41] Bacterial test strains (200 μL, 1 × 10^6^ colony-forming units (CFU) mL^−1^) were added to the Au–SAM peptide surface samples and incubated at 37 °C for 1 h to allow bacterial cells to adhere to the sample surface. Loosely bound bacteria were then removed by washing in PBS pH 7.2 for 10 min before the samples were incubated in 200 μL of minimum medium (PBS with 1% tryptic soy broth (TSB)) at 37 °C for 18 h (challenge time). After the removal of the test samples, each well was supplemented with 50 μL of TSB complete medium. The bacterial growth (of the daughter cells) at 37 °C was recorded every 30 min (readout time, Software KC4 3.4, BioTek) for a period of 48 h by OD measurements in a microtiter plate reader at a wavelength of 578 nm. The onset OD value was defined as 0.2.

#### 4.2.8. ToF-SIMS Mass Spectrometry Imaging

The coupling efficacy/homogeneity was evaluated by imaging mass spectroscopy following the presence of peptide-specific fragments on the surface. The ToF-SIMS analysis was carried out in a ToFSIMS IV instrument (IONToF GmbH, Germany), using 25 keV Bi3+ primary ions at a pulsed current of 0.1 pA (cycle time 150 µs, width 1.2 ns). Each sample was analyzed in the bunched mode at an analysis area of 500 × 500 µm^2^ (resolution of 256 pixels). The average of 25 scans at an acquisition time of 100 s was used when acquiring data.

Reference spectra of pure peptides were measured by placing a drop of peptide dissolved in EtOH at 1% (*w/v*) on a clean silica wafer. After the evaporation of the EtOH, the peptide was analyzed and specific mass fragments from each peptide were identified, such as arginine (100,09 *m/z*) and tryptophan (130, 07 *m/z*). The distribution of coupled peptides on the Au/PEG/alkyne surfaces was then determined by following the presence of these fragments.

#### 4.2.9. Contact Angle Measurements

The static water contact angle before and after peptide coupling was measured using the sessile drop method. A DSA100 instrument from Krüss GmbH (Hamburg, Germany) was used for the deposition and image evaluation of drop shape. A drop volume of 5 µL was deposited using ultrapure and deionized water (resistivity > 18.2 MΩ^−1^ cm) and the drop shape was measured 10 s after surface deposition. The analysis was performed under ambient temperature and humidity. The contact angles were obtained through ten independent measurements (Table S1).

## Figures and Tables

**Table 1:** Antimicrobial activity of eight clickable peptides against four selected bacterial reference strains.

				Antimicrobial Activity (MIC in µg/mL)			
Entry	Sequence	Net Charge	Mw	*S. aureus*	*S. epidermidis*	*E. coli*	*P. aeruginosa*
**1a**	N_3_-PEG200-RWWRF	2+	1188.49	64	32	256	256
**1b**	N_3_-PEG400-RWWRF	2+	1388.49	128	128	>256	>256
**1c**	RWWRFK(N_3_)	3+	1003.19	32	16	64	64
**1d**	c(RWWRFK(N_3_))	2+	986.16	8	8	64	128
**2a**	N_3_-PEG200-RBBRF	2+	1262.53	8	8	64	64
**2b**	N_3_-PEG400-RBBRF	2+	1462.53	32	16	128	256
**2c**	RBBRFK(N_3_)	3+	1077.31	8	4	8	16
**2d**	c(RBBRFK(N_3_))	2+	1060.28	4	2	64	256

Bacterial reference strains: Staphylococcus aureus ATCC 9144; Staphylococcus epidermidis 1457; Escherichia coli ATCC 25922; Pseudomonas aeruginosa ATCC 27853. Full data set can be found in Supplementary Information Table S3.

**Table 2:** The average contact angles θ (standard deviation) of **1a**–**d**, **2a**–**d** covalently linked to Au surface. Control is Au surface treated with peptide, without the CuAAC reaction.

1a	1b	1c	1d	2a	2b	2c	2d	Control
49.1 (2.7)	52.2 (2.5)	50.0 (3.2)	49.6 (2.1)	54.7 (2.4)	53.5 (2.3)	54.9 (3.4)	54.0 (2.5)	39.6 (2.8)

## Data Availability

The data presented in this study are available in Supplementary Material.

## Supplementary Materials

The following are available online at https://www.mdpi.com/article/10.3390/antibiotics10121516/s1, Figures S1–S8: ^1^H NMR spectra of compounds **3a** and **b**, **4a** and **b**, **5a** and **b**, and **6a** and **b**, Figures S9–S16: ^1^H NMR spectra of compounds **1a**–**d**, and **2a**–**d**, Figures S17–S24: HPLC of compounds **1a**–**d**, and **2a**–**d**, Figure S25: Contact angle images of **1a**–**d** and **2a**–**d** covalently linked to an Au surface, Figure S26: ToF-SIMS images of **1a**–**d** covalently linked to an Au surface, Figure S27: ToF-SIMS images of **2a**–**d** covalently linked to an Au surface, Table S1: Contact Angle of **1a**–**d** and **2a**–**d** covalently linked to an Au surface, Table S2: Certika data of **1a**–**d** and **2a**–**d** covalently linked to an Au surface, Table S3: Overview of MIC data.
