# Supplementary material for: Anti-Colonization Effect of Au Surfaces with Self-Assembled Molecular Monolayers Functionalized with Antimicrobial Peptides on S. epidermidis
Source: Antibiotics (Basel). 2021 Dec 10;10(12):1516. doi: 10.3390/antibiotics10121516 (PMC8698454; doi:10.3390/antibiotics10121516)
Supplement: Supplementary file 1 [file antibiotics-10-01516-s001.zip › antibiotics-1507120-supplementary.pdf]

Anti-colonization Effect of Au Surfaces with Self-Assembled Molecular Monolayers Functionalized with Antimicrobial Peptides on *S. Epidermidis*

Eskil André Karlsen [1,2], Wenche Stensen [1,2], Eric Juskewitz [3], Johan Svenson [4], Mattias Berglin [4] and John Sigurd Mjøen Svendsen [1,2,*]

[1] Amicoat AS, Sykehusvegen 23, 9019 Tromsø, Norway; eskil.a.karlsen@uit.no (E.A.K.); wenche.stensen@uit.no (W.S.)
[2] Department of Chemistry, Faculty of Science and Technology, UiT—The Arctic University of Norway, NO-9037 Tromsø, Norway
[3] Department of Medical Biology, Faculty of Health Sciences, UiT—The Arctic University of Norway, NO-9037 Tromsø, Norway; eric.juskewitz@uit.no (E.J.)
[4] RISE Research Institutes of Sweden, Brinellgatan 4, 504 62 Borås, Sweden; mattias.berglin@ri.se (M.B.); johan.svenson@cawthron.org.nz (J.S.)
* Correspondence: john-sigurd.svendsen@uit.no (J.S.M.S.); Tel.: +47-7764-4086

^{1}H NMR of *O,O'*-bis(tosyloxy)polyethylene glycols

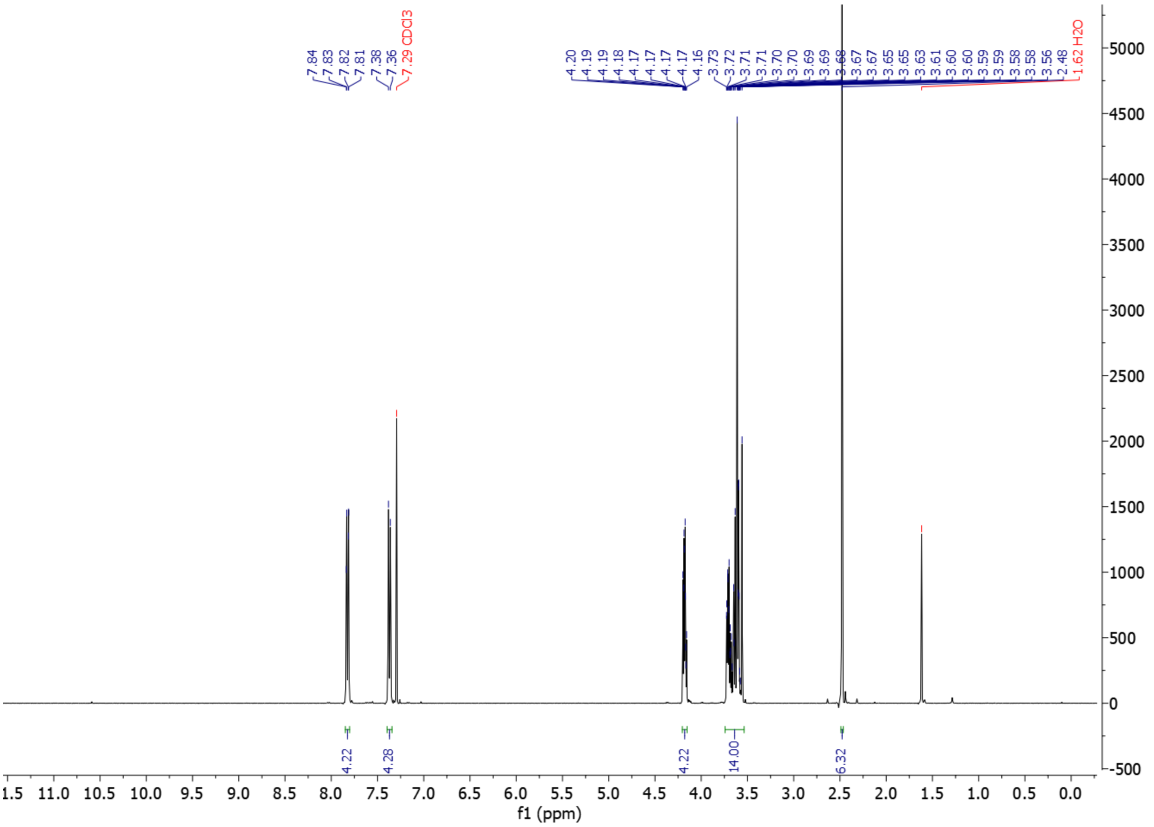

Figure S1. ^{1}H NMR of compound **3a**.

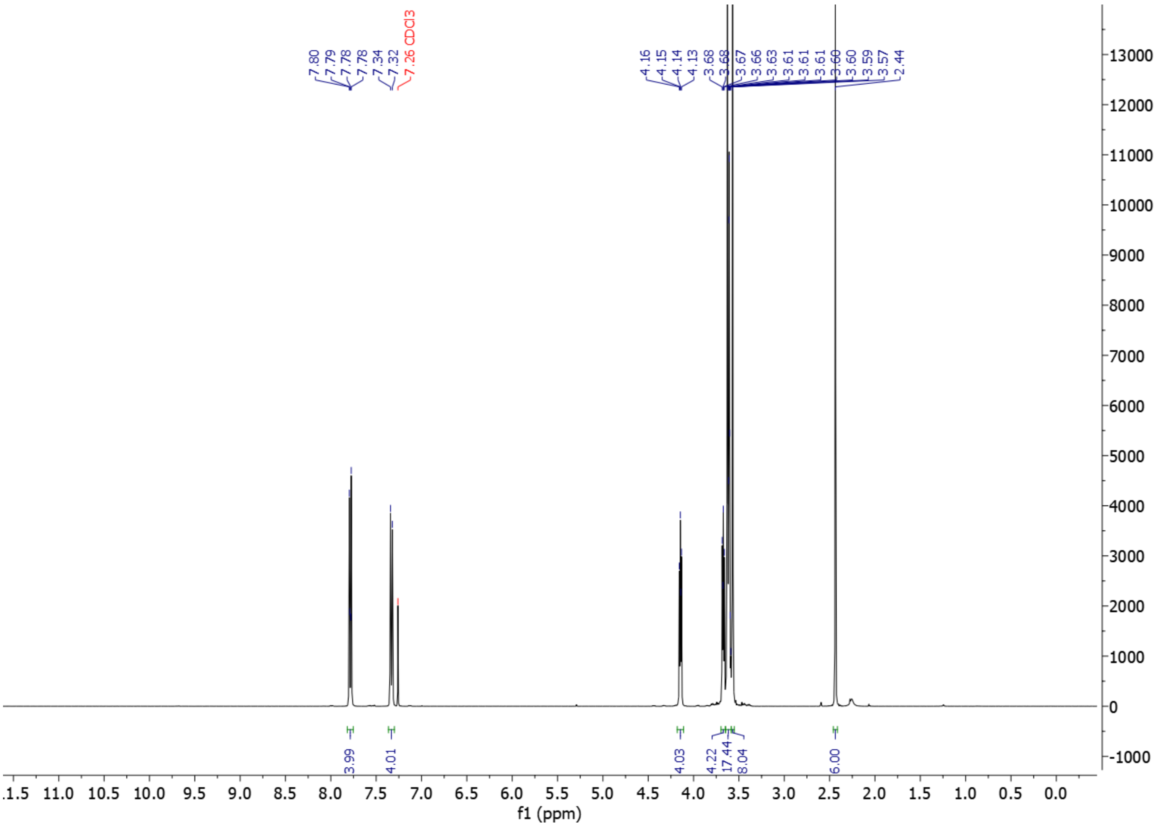

Figure S2. ^{1}H NMR of compound **3b**.

^{1}H NMR of *O,O′*-bis(2-azidoethyl)polyethylene glycols

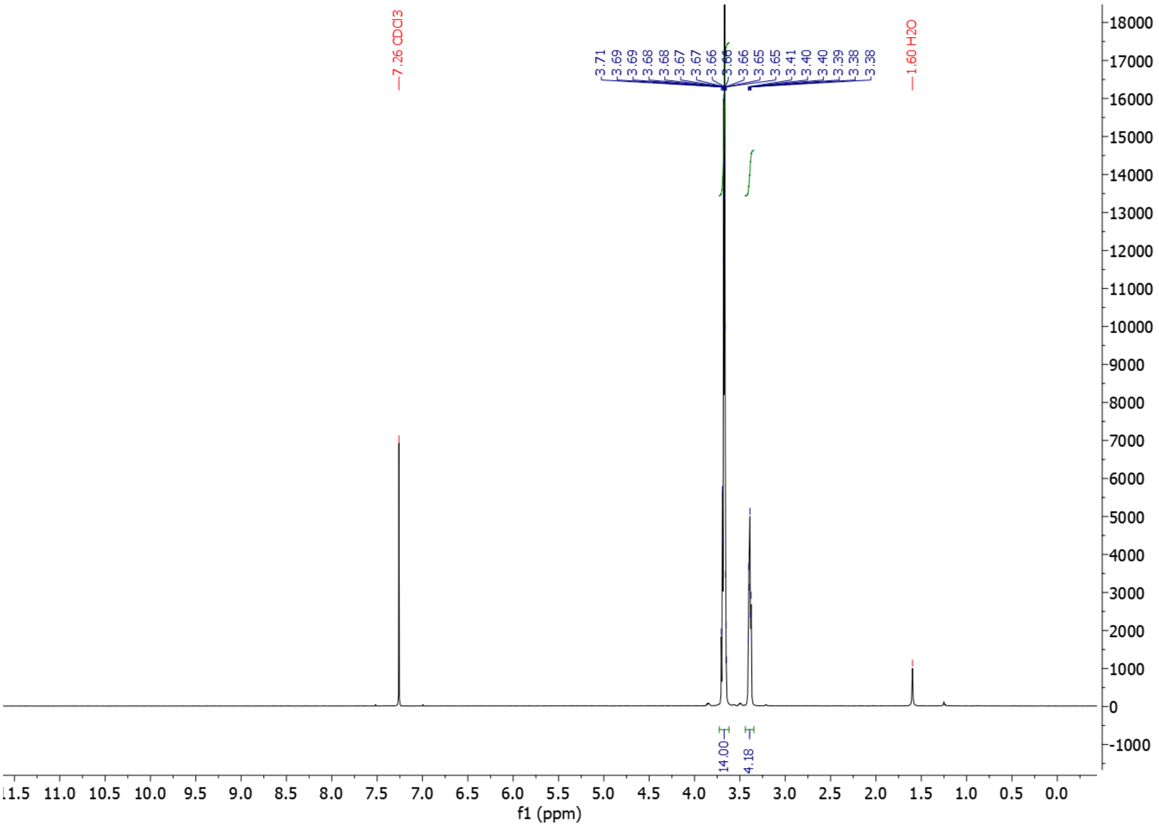

Figure S3. ^{1}H NMR of compound **4a**.

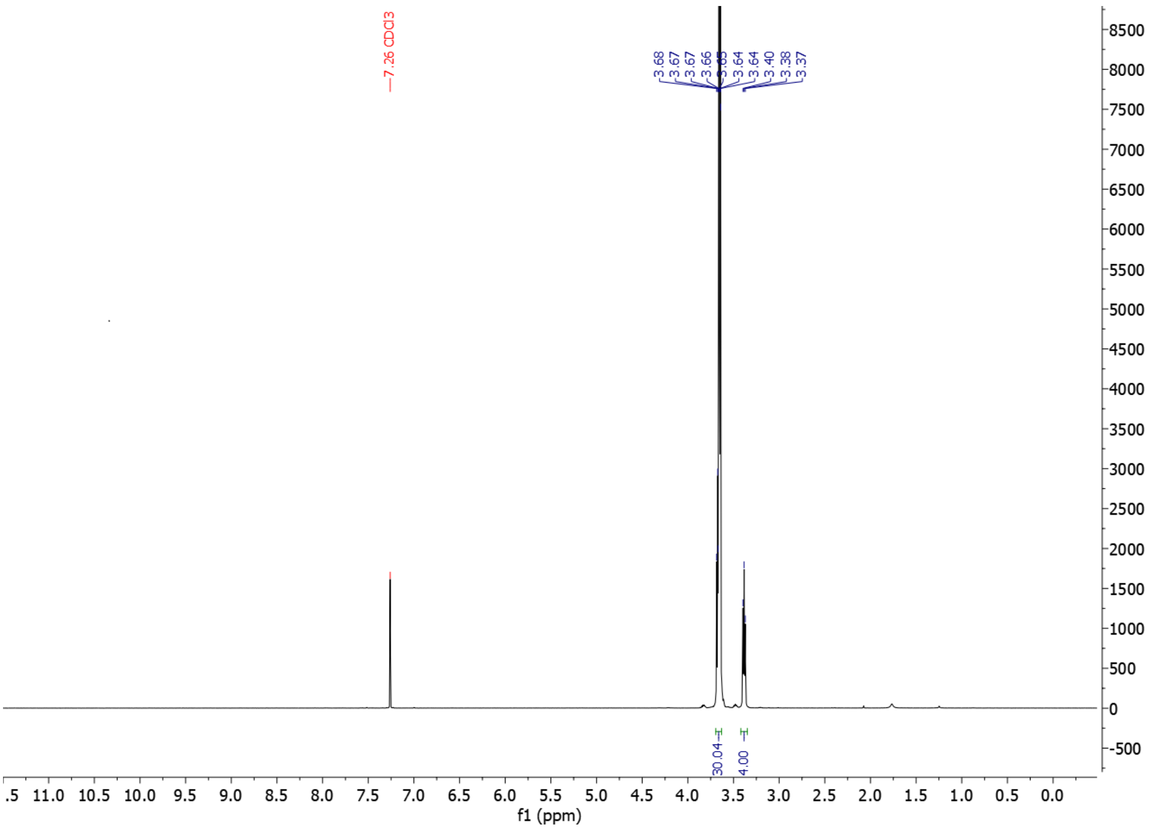

Figure S4. ^{1}H NMR of compound **4b**.

^{1}H NMR of *O*-(2-aminoethyl)-*O′*-(2-azidoethyl)polyethylene glycols

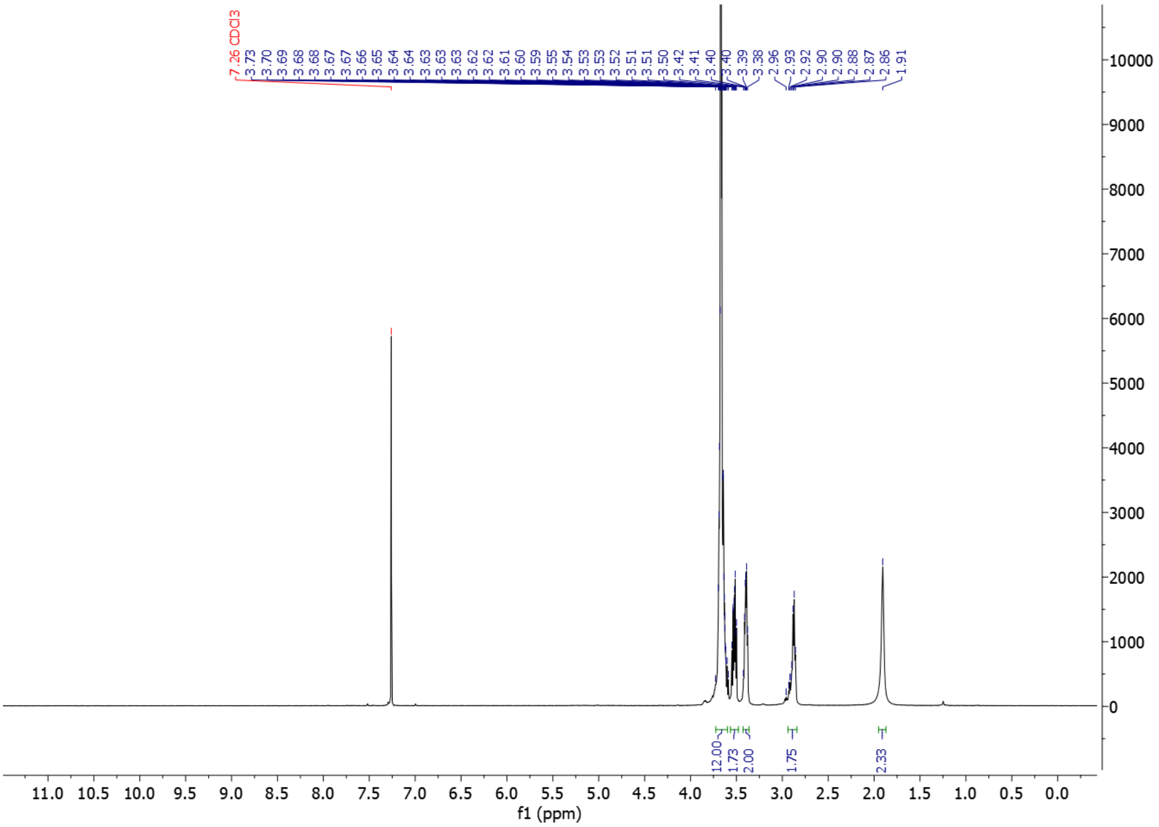

Figure S5. ^{1}H NMR of compound **5a**.

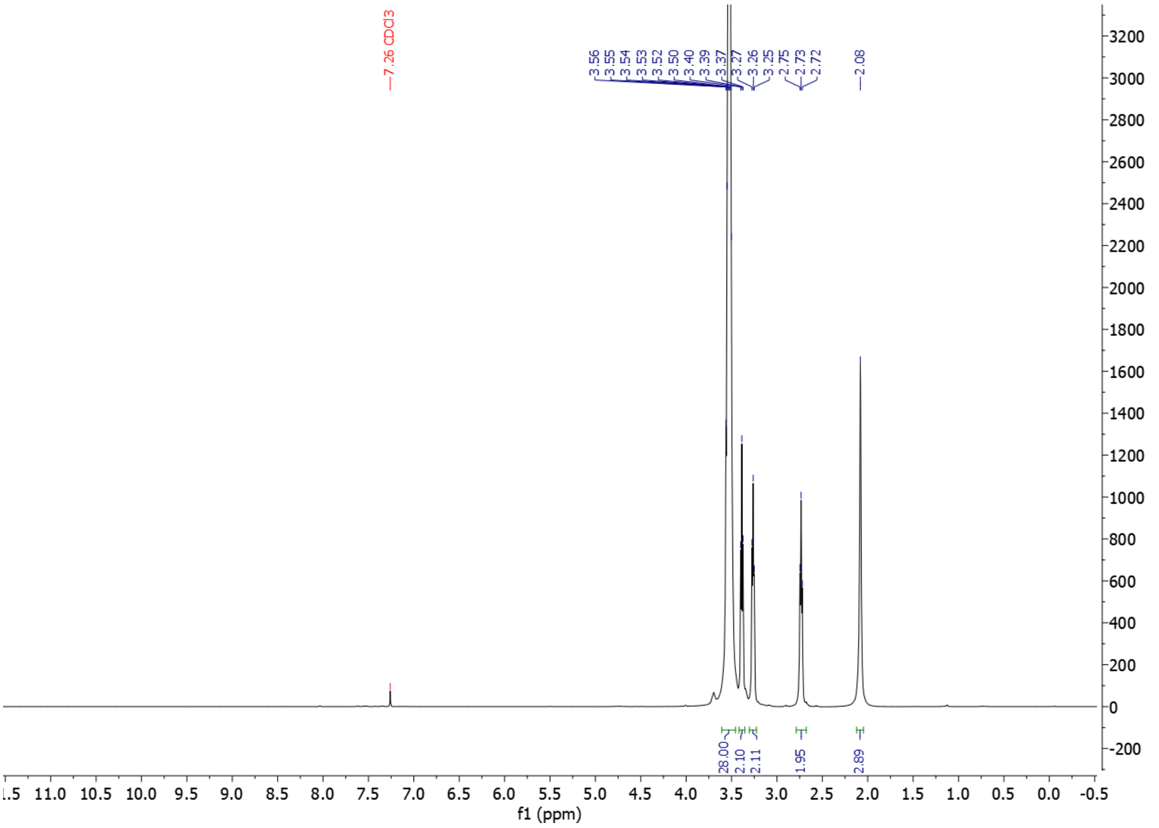

Figure S6. ^{1}H NMR of compound **5b**.

^{1}H NMR of *O*-(2-azidoethyl)-*O*-[2-(diglycolyl-amino)ethyl]polyethylene glycols

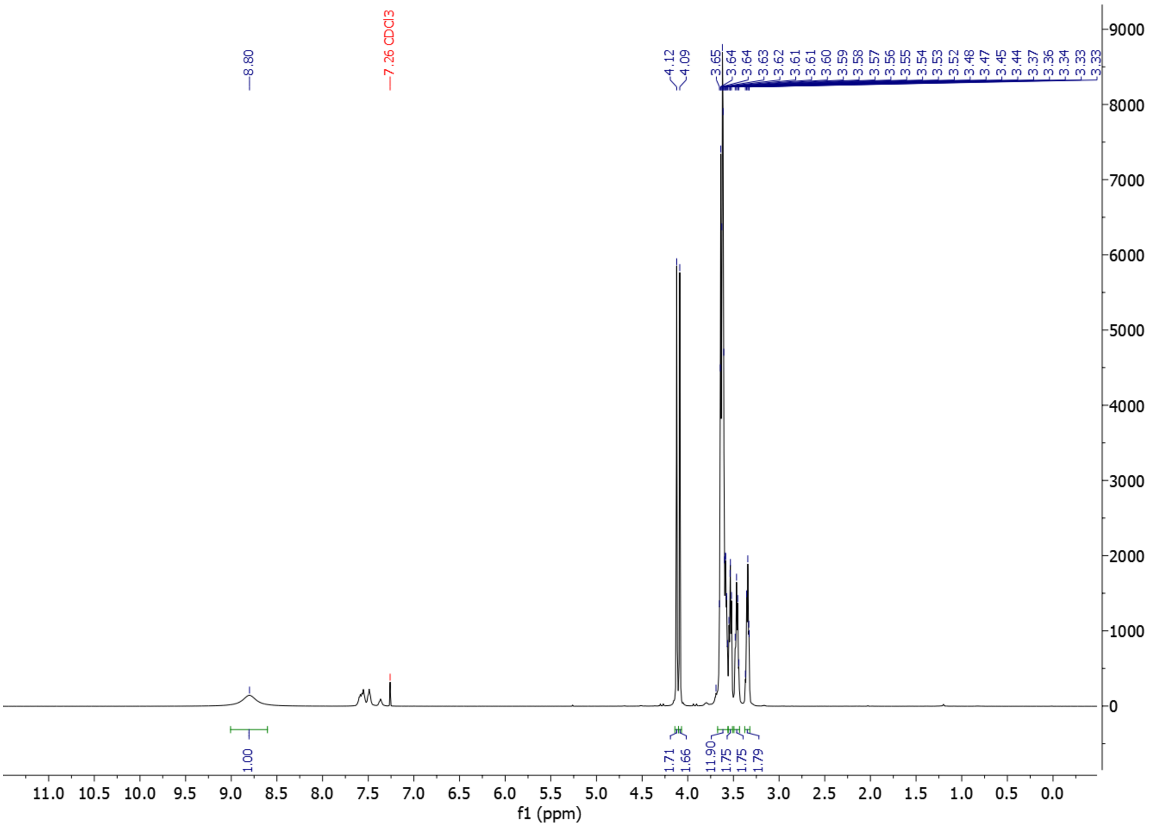

Figure S7. ^{1}H NMR of compound **6a**.

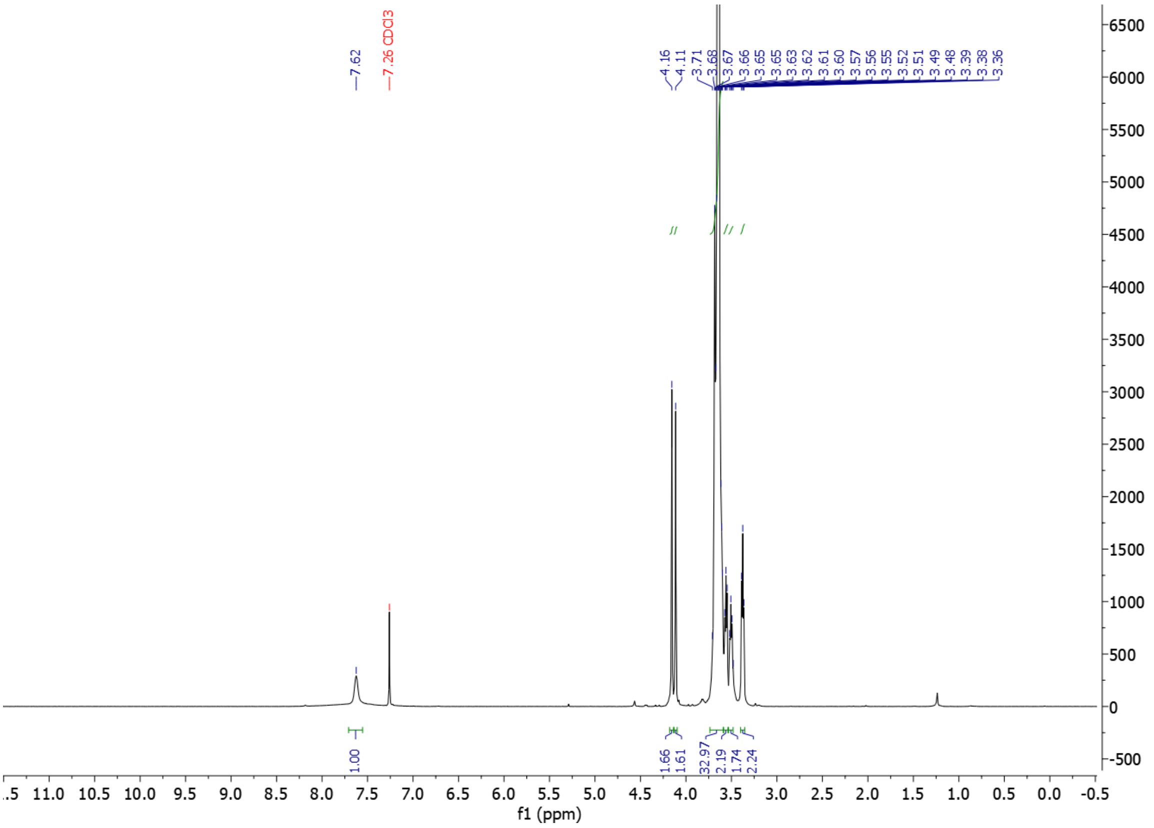

Figure S8. ^{1}H NMR of compound **6b**.

^{1}H NMR of azidopeptides

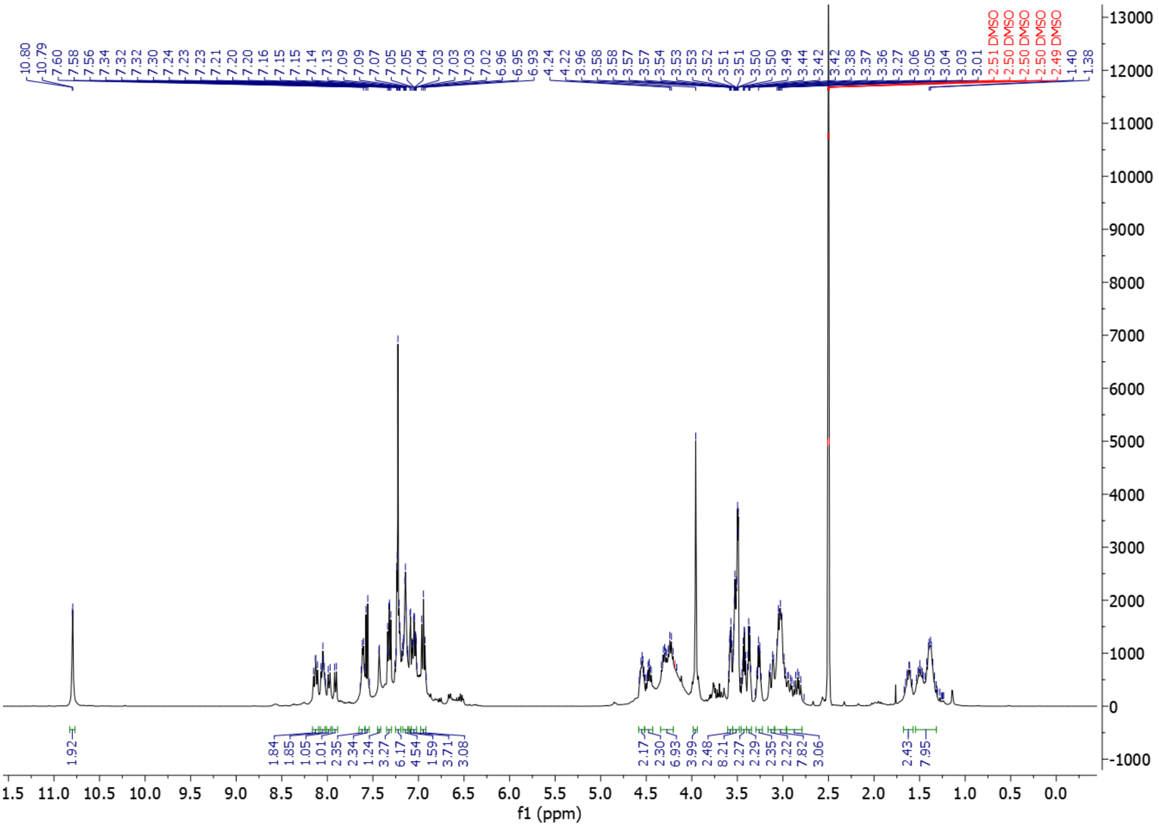

Figure S9. ^{1}H NMR of compound **1a**.

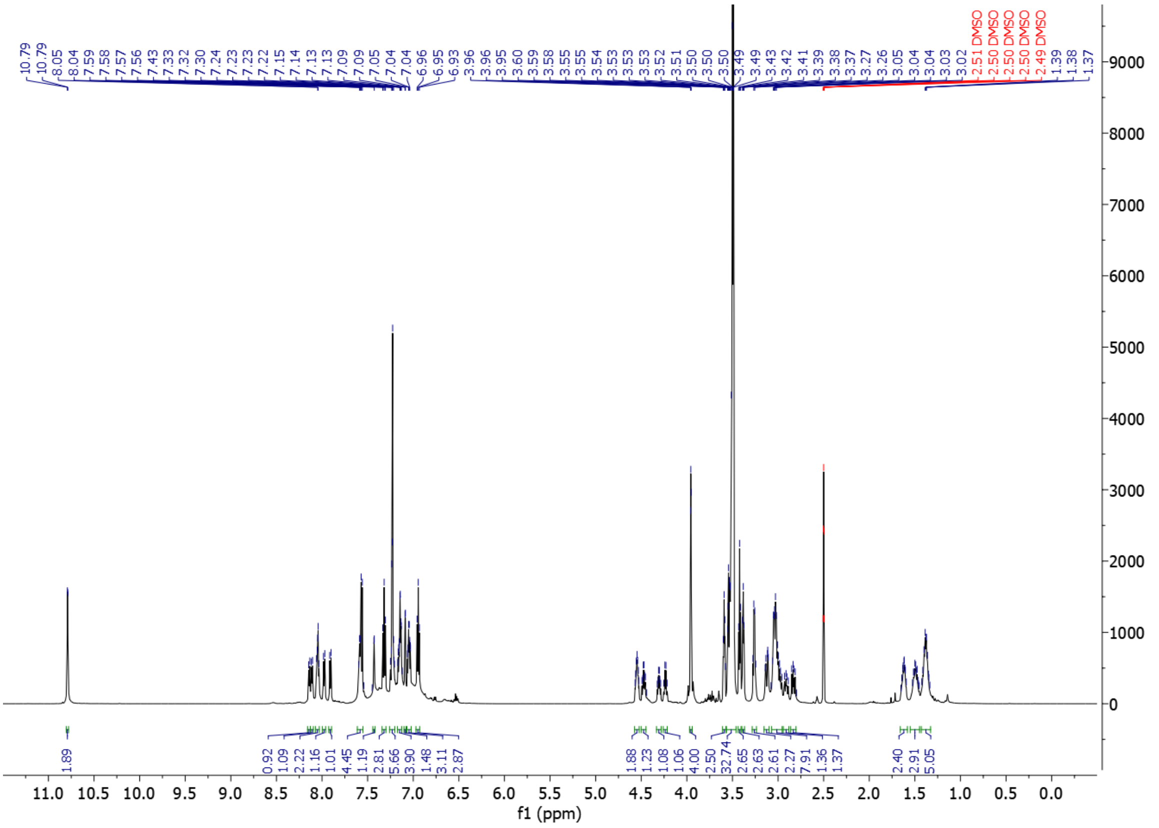

Figure S10. ^{1}H NMR of compound **1b**.

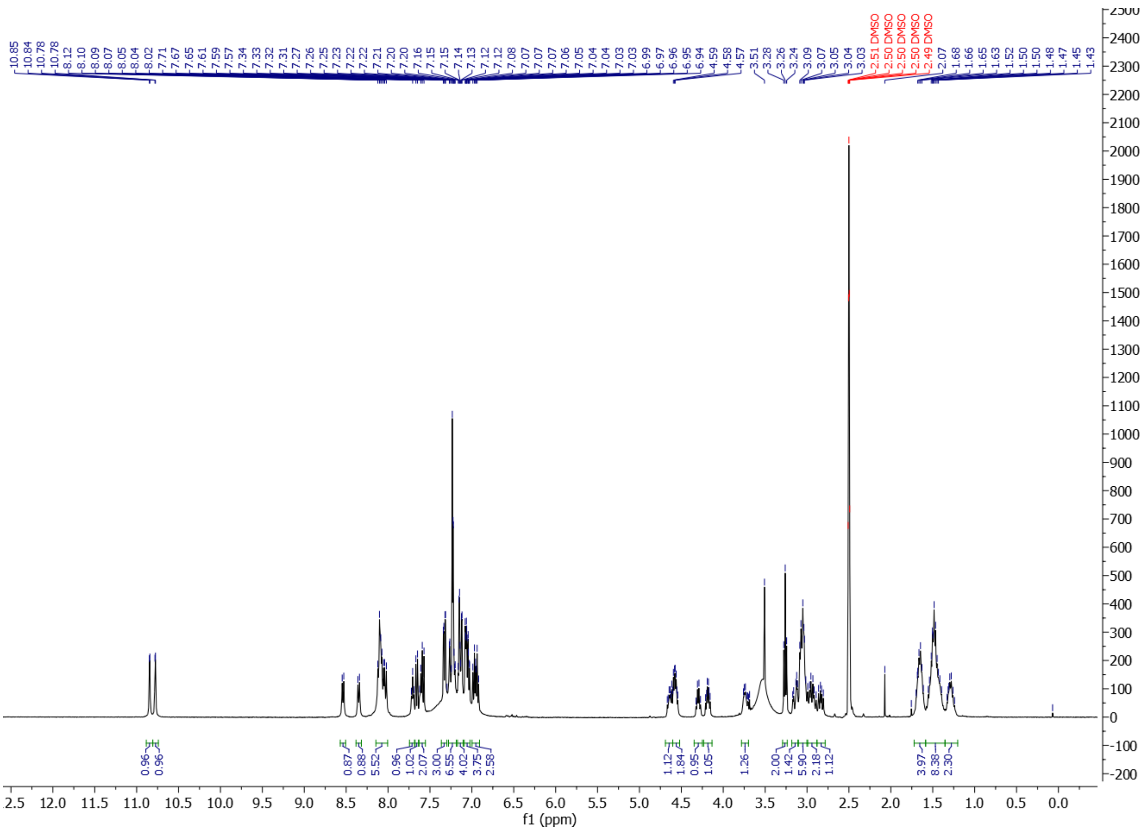

Figure S11. ^{1}H NMR of compound **1c**.

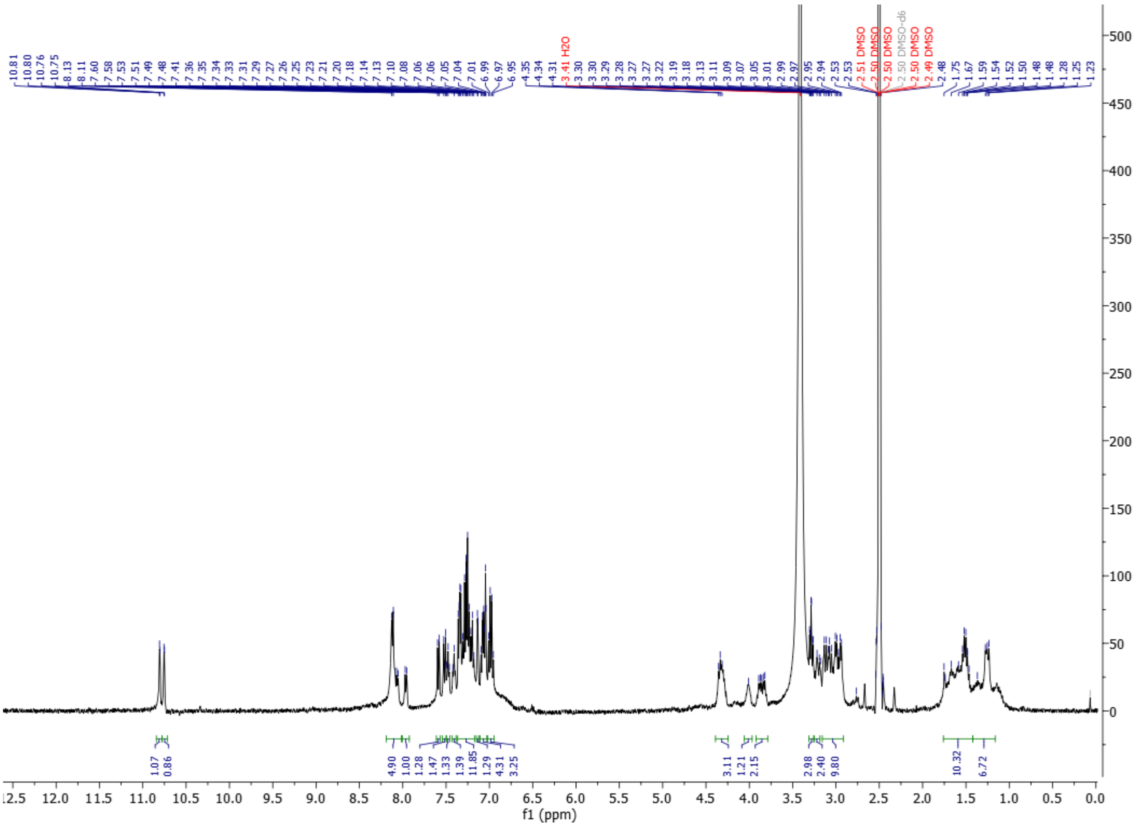

Figure S12. ^{1}H NMR of compound **1d**.

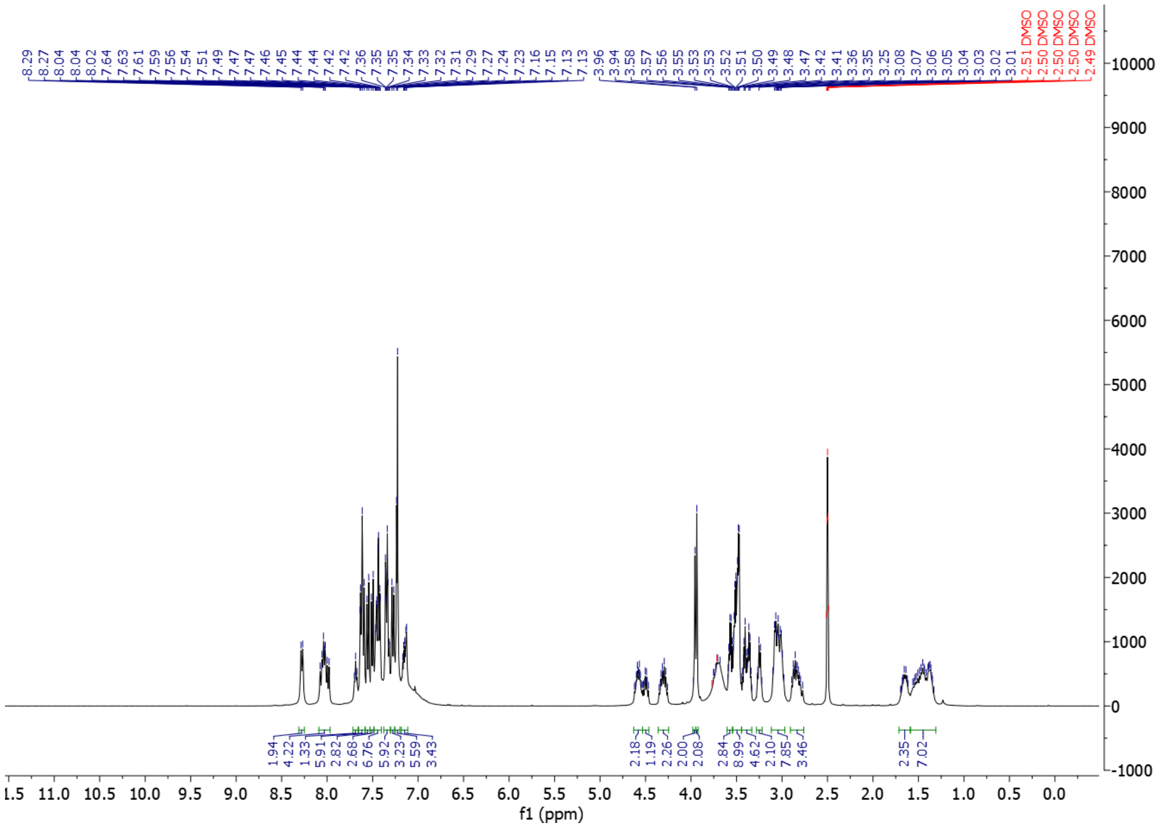

Figure S13. ^{1}H NMR of compound **2a**.

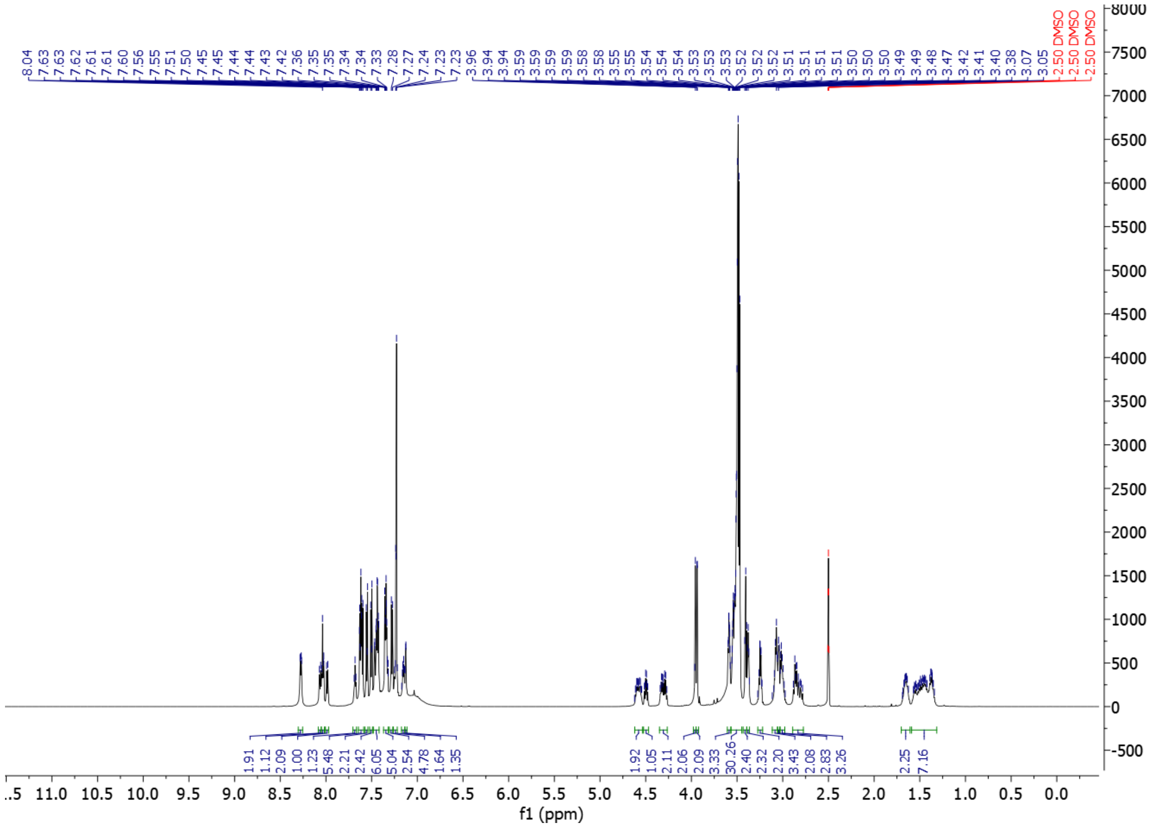

Figure S14. ^{1}H NMR of compound **2b**.

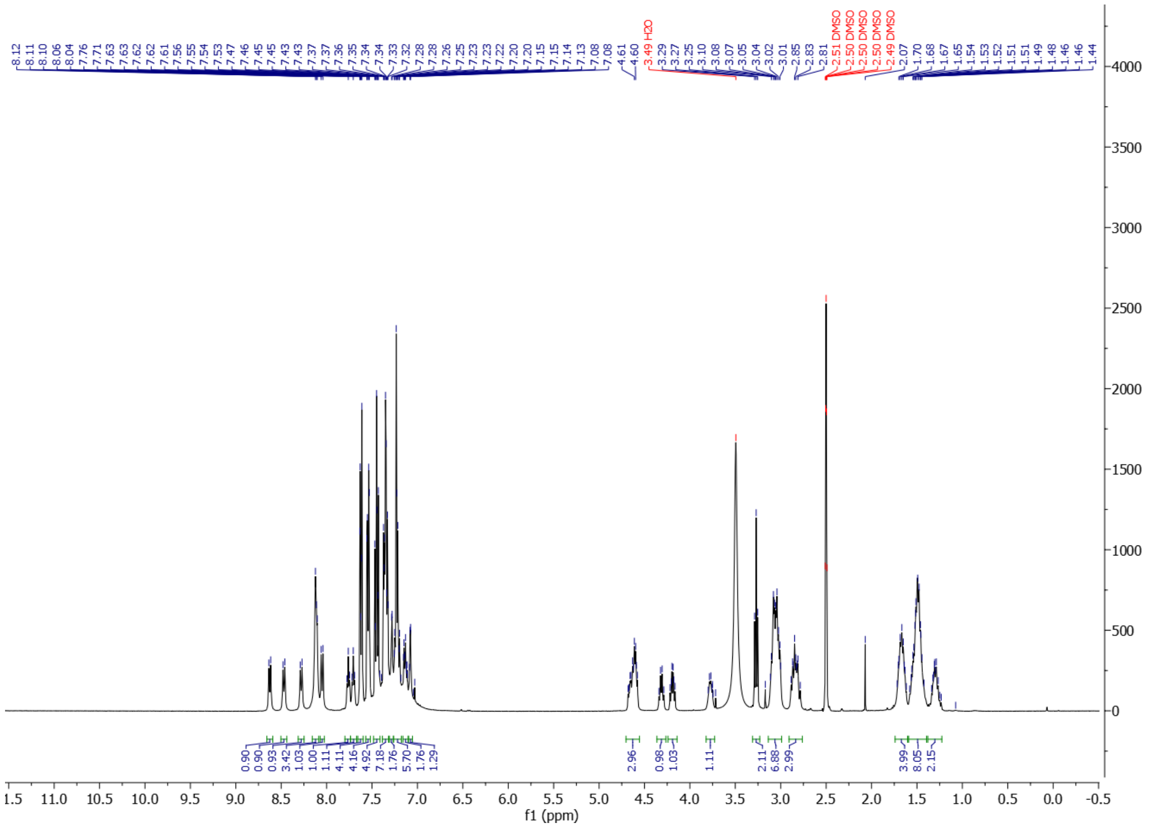

Figure S15. ^{1}H NMR of compound **2c**.

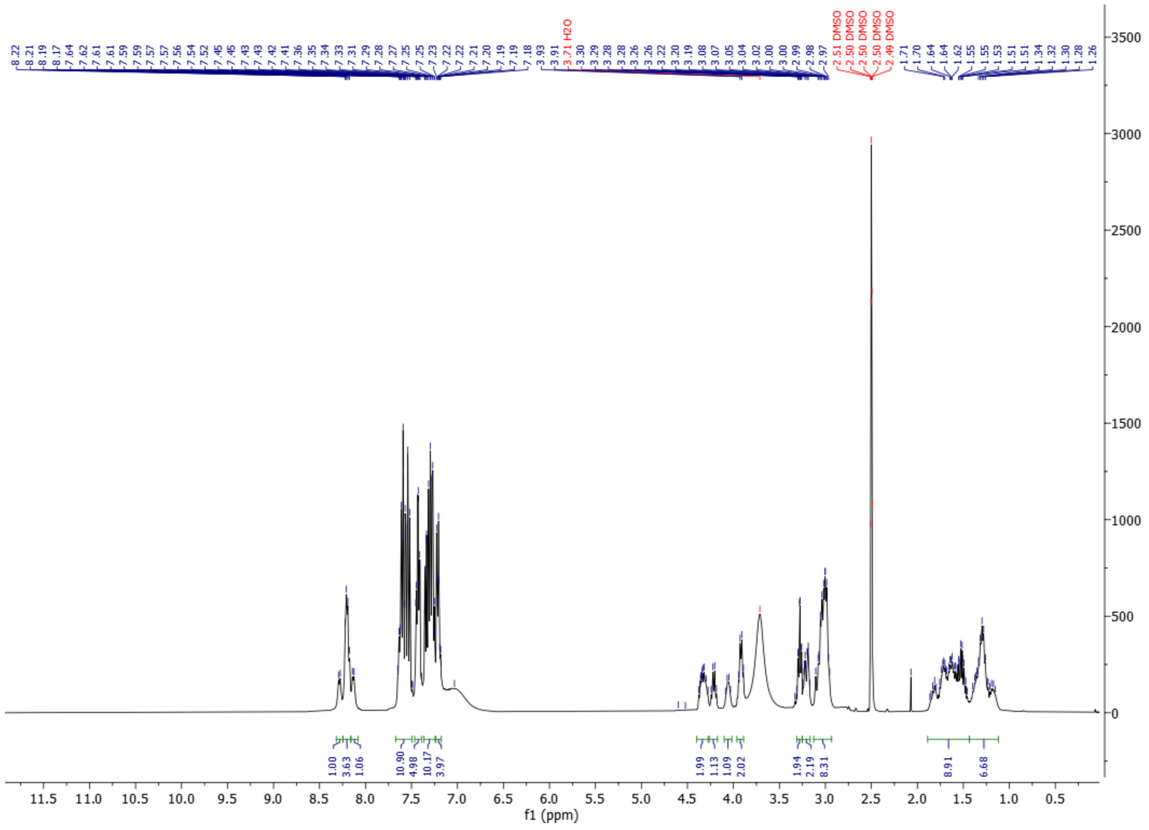

Figure S16. ^{1}H NMR of compound **2d**.

Analytical HPLC of azidopeptides

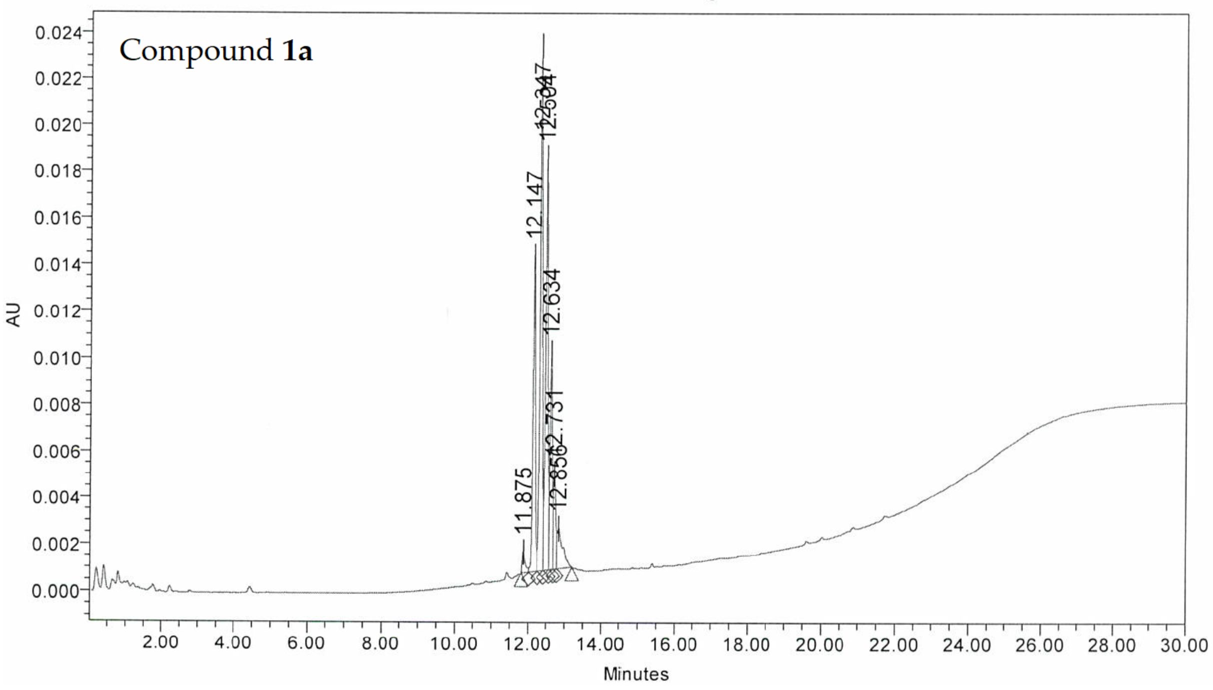

Figure S17. Analytical HPLC of compound **1a**.

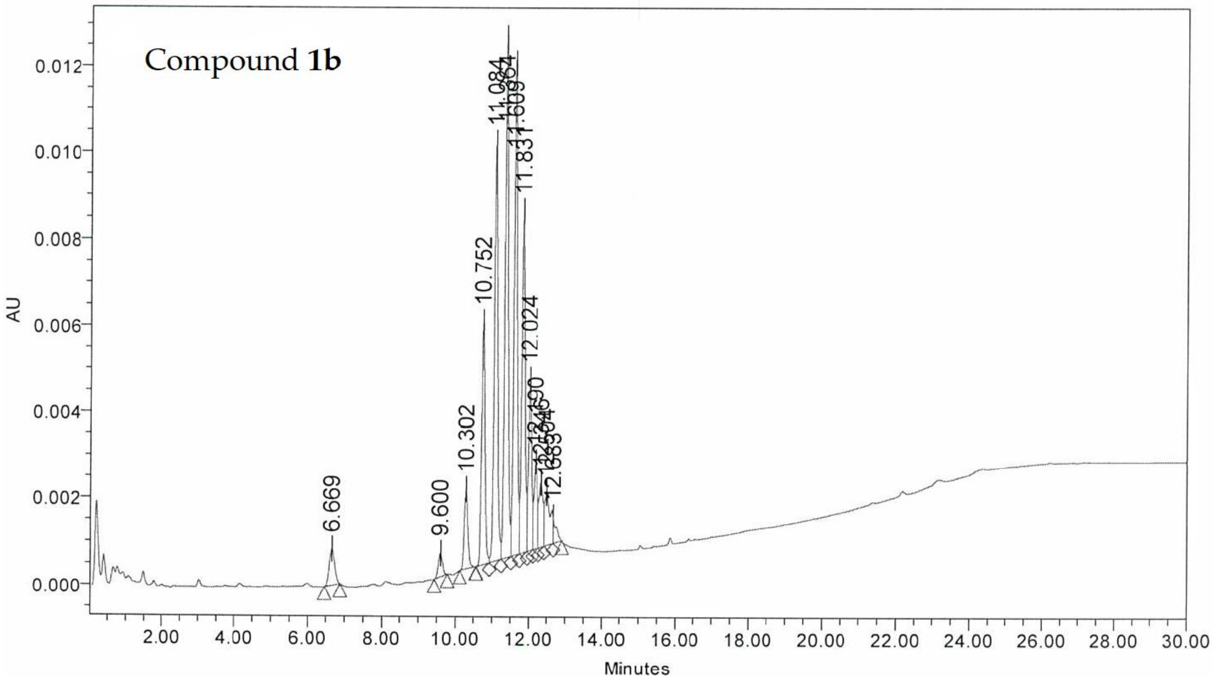

Figure S18. Analytical HPLC of compound **1b**.

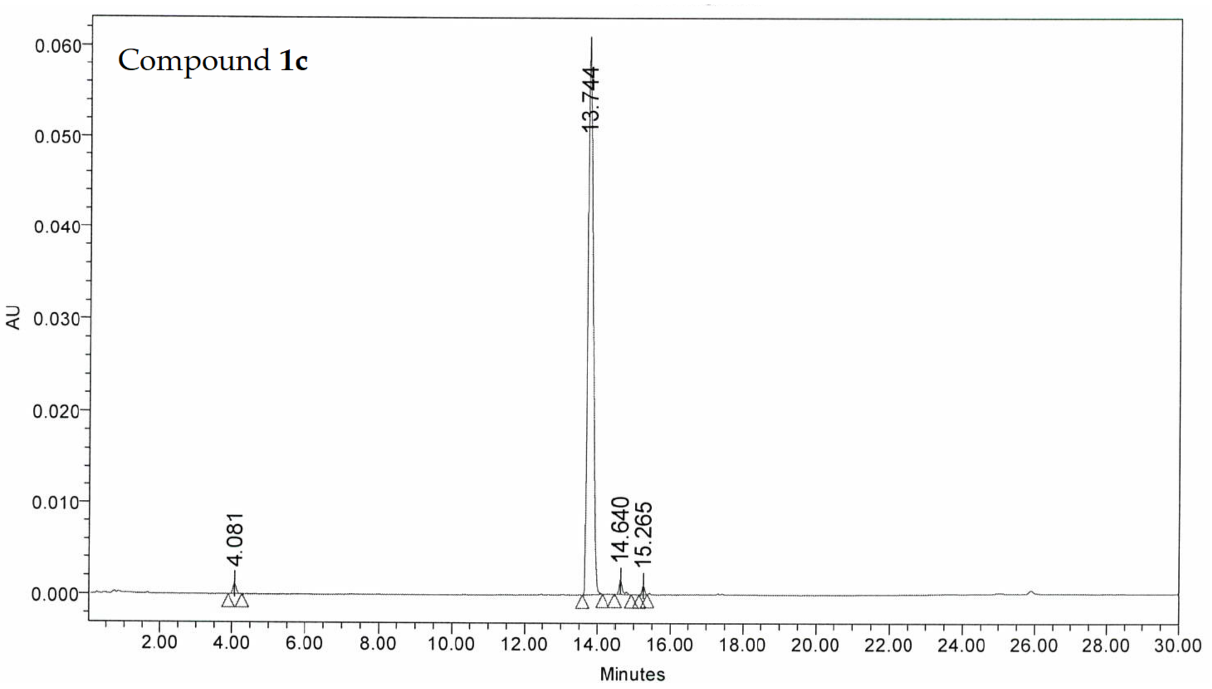

Figure S19. Analytical HPLC of compound **1c**.

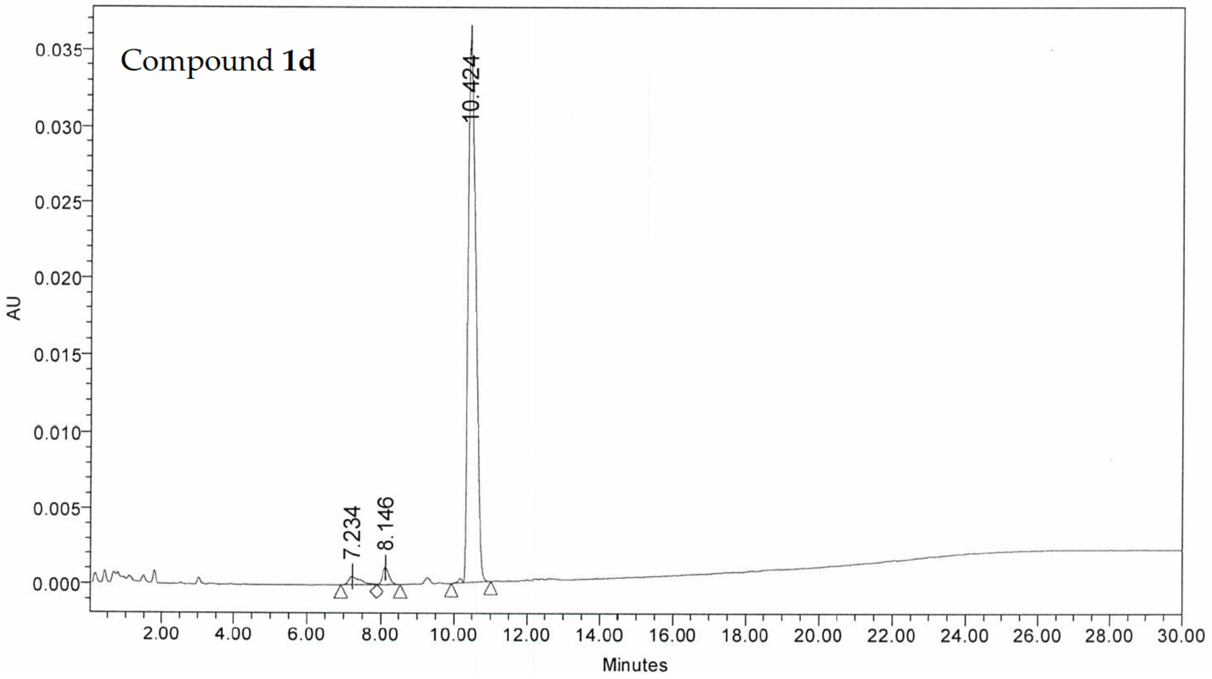

Figure S20. Analytical HPLC of compound **1d**.

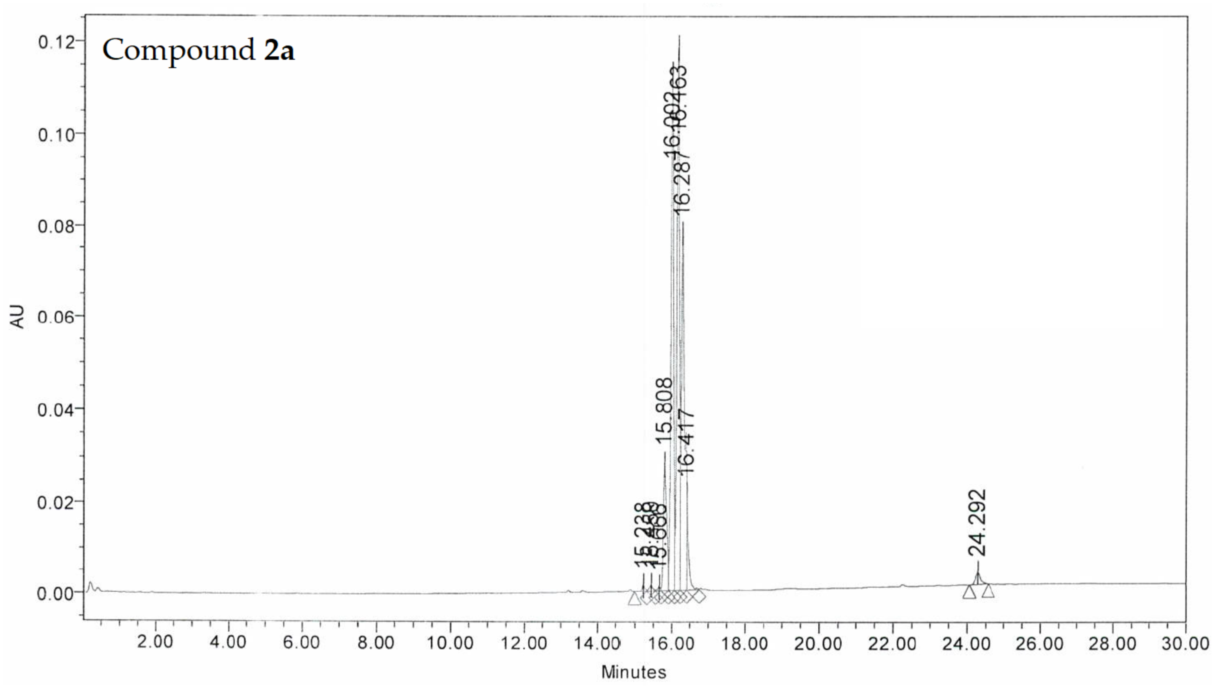

Figure S21. Analytical HPLC of compound **2a**.

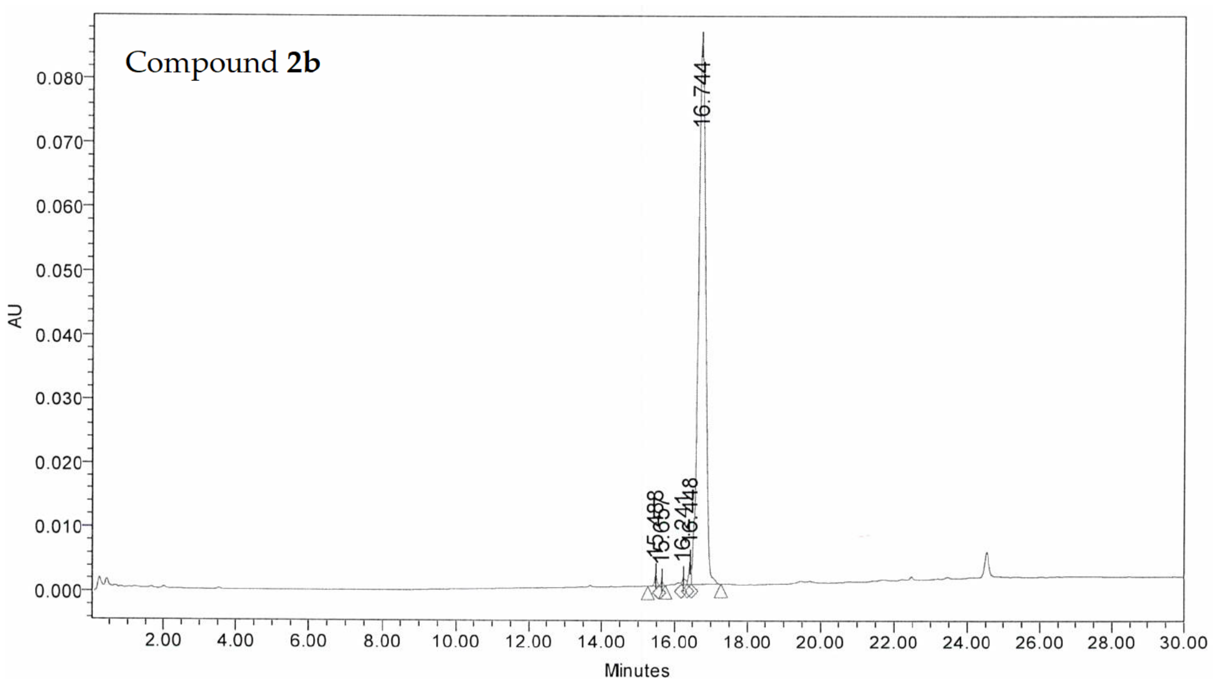

Figure S22. Analytical HPLC of compound **2b**.

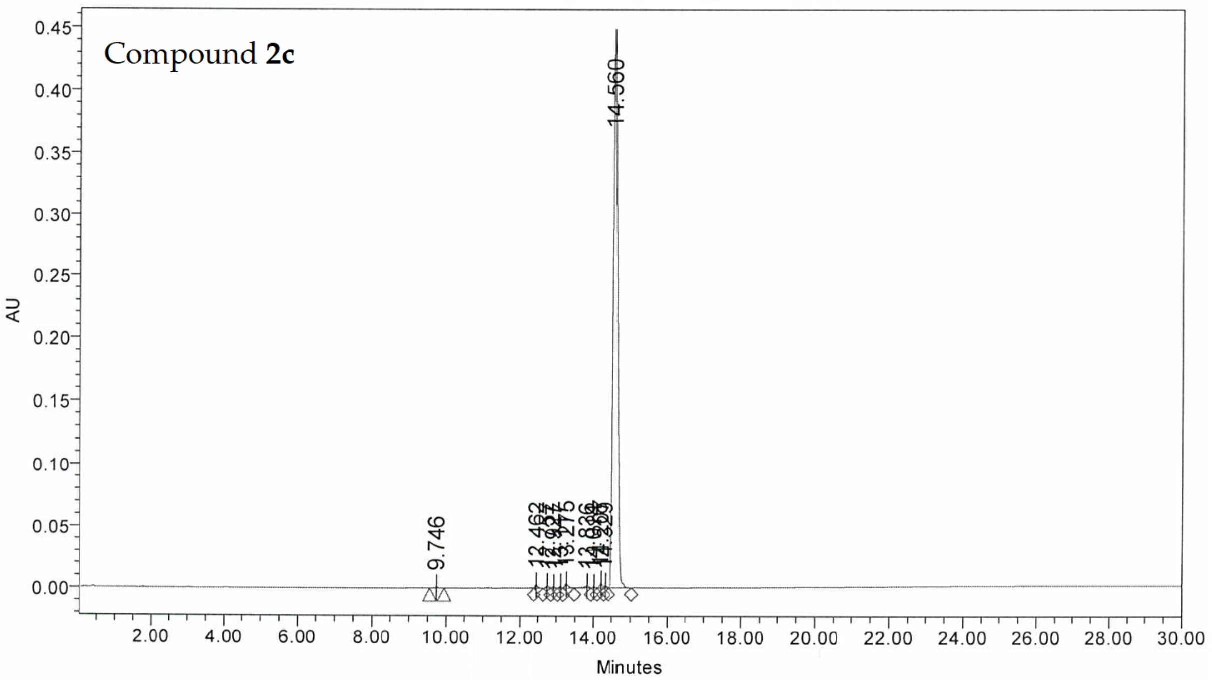

Figure S23. Analytical HPLC of compound **2c**.

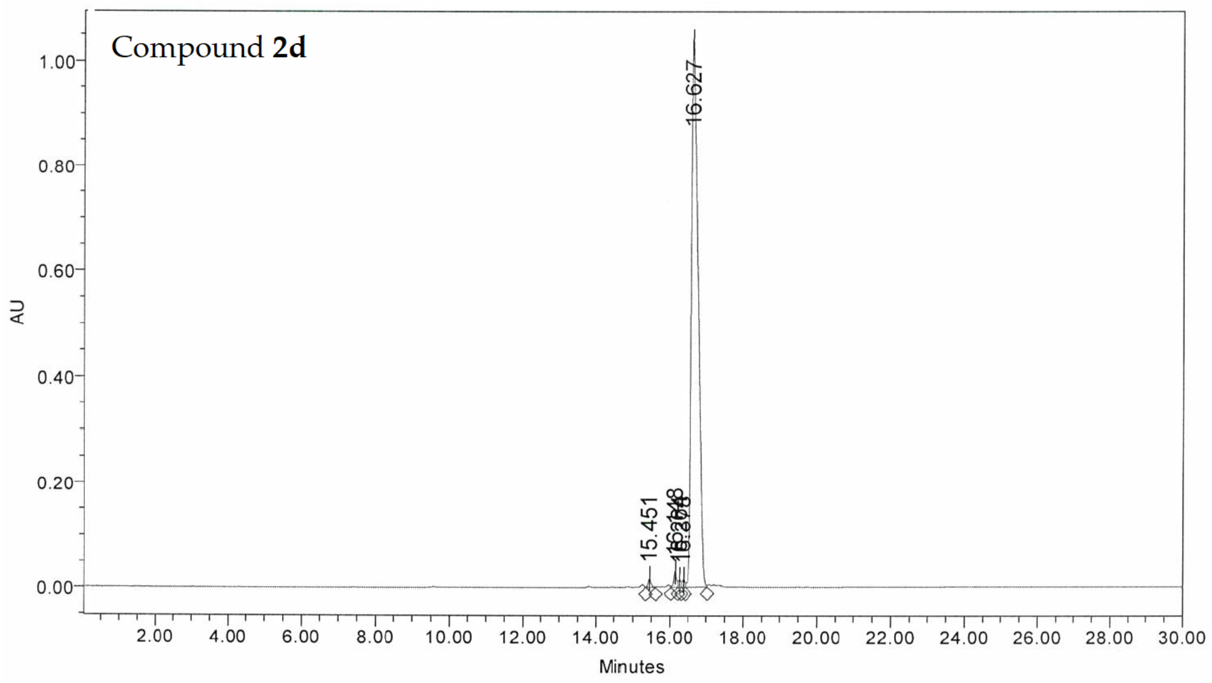

Figure S24. Analytical HPLC of compound **2d**.

Contact angle data

Table S1. Contact Angle of **1a–d** and **2a–d** covalently linked to an Au surface. Control is Au surface treated with peptide, without the CuAAC reaction

No.	θ(M) [deg]	θ(L) [deg]	θ(R) [deg]	Fit-Er [um]	Method	Volum [ul]	BD [mm]
Au_1a_1	45.6 ± 0.06	45.5	45.6	0.92	T-1	0.211	1.349
Au_1a_2	52.1 ± 0.41	52.5	51.7	1.61	T-1	0.301	1.446
Au_1a_3	51.8 ± 0.66	51.1	52.4	2.28	T-1	0.249	1.368
Au_1a_4	48.2 ± 0.92	47.3	49.1	1.28	T-1	0.309	1.502
Au_1a_5	48.0 ± 1.10	46.9	49.1	1.06	T-1	0.260	1.422
Au_1b_1	56.2 ± 0.96	55.3	57.2	0.97	T-1	0.288	1.372
Au_1b_2	52.7 ± 0.39	53.1	52.3	1.16	T-1	0.269	1.372
Au_1b_3	50.6 ± 1.17	51.8	49.5	1.14	T-1	0.266	1.405
Au_1b_4	50.3 ± 0.86	49.4	51.1	0.88	T-1	0.277	1.420
Au_1b_5	51.0 ± 0.41	50.6	51.4	0.86	T-1	0.318	1.475
Au_1c_1	44.9 ± 0.60	44.3	45.5	1.10	T-1	0.280	1.514
Au_1c_2	50.5 ± 0.43	50.0	50.9	0.93	T-1	0.263	1.395
Au_1c_3	49.6 ± 0.29	49.9	49.3	1.76	T-1	0.286	1.491
Au_1c_4	54.2 ± 0.09	54.2	54.1	1.24	T-1	0.252	1.333
Au_1c_5	50.8 ± 0.23	50.6	51.0	1.15	T-1	0.264	1.383
Au_1d_1	46.5 ± 0.09	46.4	46.6	0.82	T-1	0.284	1.488
Au_1d_2	50.5 ± 0.28	50.8	50.3	1.39	T-1	0.315	1.438
Au_1d_3	49.1 ± 0.63	48.4	49.7	0.94	T-1	0.265	1.417
Au_1d_4	52.3 ± 1.26	51.1	53.6	1.54	T-1	0.168	1.184
Au_1d_5	49.6 ± 0.18	49.8	49.4	0.92	T-1	0.233	1.350
Au_2a_1	56.2 ± 0.22	56.0	56.4	0.98	T-1	0.317	1.411
Au_2a_2	50.6 ± 0.42	50.2	51.0	1.25	T-1	0.285	1.439
Au_2a_3	54.9 ± 0.92	55.9	54.0	1.10	T-1	0.304	1.413
Au_2a_4	54.7 ± 0.76	55.5	54.0	0.82	T-1	0.275	1.369
Au_2a_5	56.8 ± 1.09	57.8	55.7	0.91	T-1	0.295	1.379
Au_2b_1	52.7	52.6	52.7	1.42	T-1	0.282	1.405
Au_2b_2	53.9 ± 0.67	54.5	53.2	1.19	T-1	0.321	1.451
Au_2b_3	50.4 ± 0.95	49.5	51.4	1.18	T-1	0.285	1.429
Au_2b_4	53.7 ± 0.54	53.2	54.2	0.74	T-1	0.342	1.479
Au_2b_5	56.9 ± 0.65	56.3	57.6	0.65	T-1	0.275	1.343
Au_2c_1	57.2 ± 0.93	56.3	58.1	1.24	T-1	0.283	1.347
Au_2c_2	56.3 ± 0.59	55.7	56.9	1.08	T-1	0.335	1.442
Au_2c_3	52.6 ± 0.25	52.9	52.4	1.41	T-1	0.309	1.452
Au_2c_4	50.0 ± 0.26	50.3	49.7	1.09	T-1	0.294	1.452
Au_2c_5	58.4 ± 1.00	57.4	59.4	1.05	T-1	0.282	1.345
Au_2d_1	52.4 ± 0.71	51.7	53.2	1.15	T-1	0.251	1.347
Au_2d_2	50.8 ± 0.72	50.1	51.5	0.77	T-1	0.288	1.429
Au_2d_3	56.9 ± 0.11	56.8	57.0	0.64	T-1	0.267	1.322
Au_2d_4	56.4 ± 0.18	56.2	56.6	1.00	T-1	0.298	1.385
Au_2d_5	53.3 ± 0.25	53.1	53.6	0.67	T-1	0.288	1.402
Au_Control_1	36.1 ± 0.63	35.5	36.8	1.02	T-1	0.225	1.541
Au_Control_2	40.0 ± 1.75	38.3	41.8	0.76	T-1	0.250	1.510
Au_Control_3	41.4 ± 1.20	40.2	42.6	1.04	T-1	0.234	1.473
Au_Control_4	37.7 ± 1.03	38.7	36.7	1.46	T-1	0.303	1.643
Au_Control_5	42.8 ± 0.27	42.5	43.0	0.92	T-1	0.208	1.372

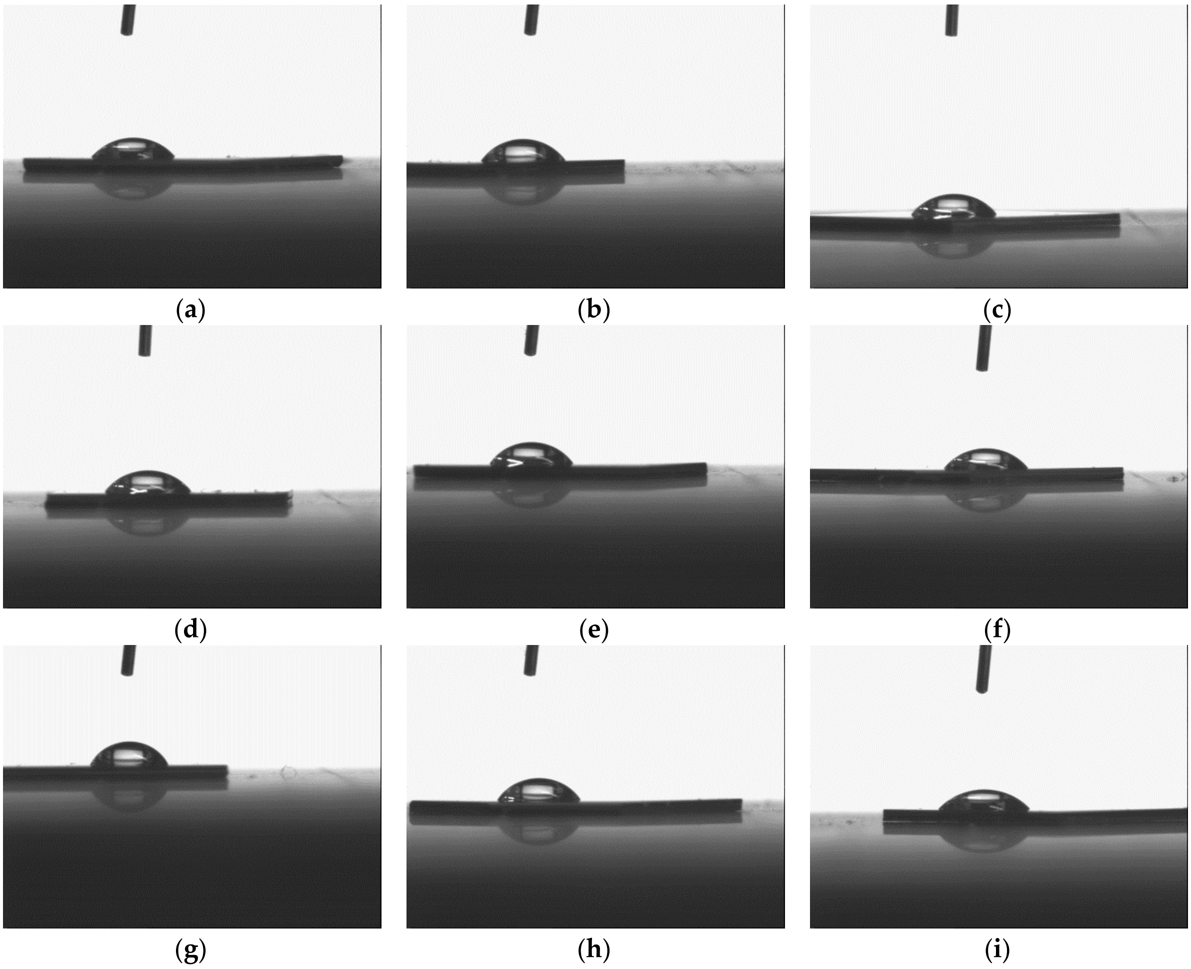

Figure S25. Contact angle images of **1a**–**d** and **2a**–**d** covalently linked to an Au surface. Control is Au surface treated with peptide, without the CuAAC reaction: (**a**) **1a** on Au surface; (**b**) **1b** on Au surface; (**c**) **1c** on Au surface; (**d**) **1d** on Au surface; (**e**) **2a** on Au surface**;** (**f**) **2b** on Au surface; (**g**) **2c** on Au surface; (**h**) **2d** on Au surface; (**i**) Au surface as a control group.

ToF-SIMS of series 1 on Au surfaces

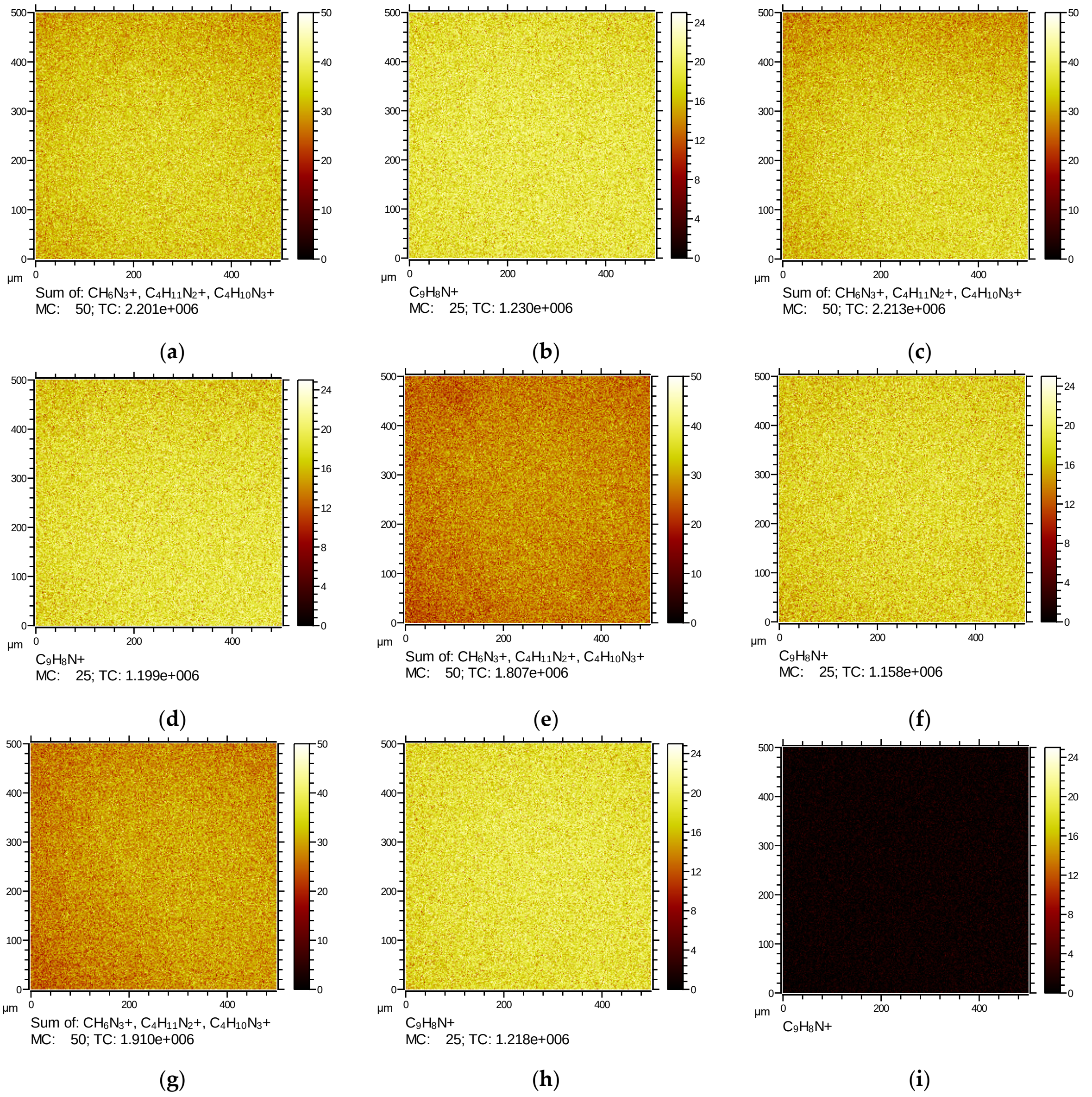

Figure S26. ToF-SIMS images of series 1 containing tryptophan: (**a**) **1a** surface of arginine residues with ion intensities for $CH_6N_3^+$, $C_4H_{11}N_2^+$, and $C_4H_{10}N_3^+$; (**b**) **1a** surface of tryptophan residues with ion intensity for $C_9H_8N^+$; (**c**) **1b** surface of arginine residues with ion intensities for $CH_6N_3^+$, $C_4H_{11}N_2^+$, and $C_4H_{10}N_3^+$; (**d**) **1b** surface of tryptophan residues with ion intensity for $C_9H_8N^+$; (**e**) **1c** surface of arginine residues with ion intensities for $CH_6N_3^+$, $C_4H_{11}N_2^+$, and $C_4H_{10}N_3^+$; (**f**) **1c** surface with tryptophan residues with ion intensity for $C_9H_8N^+$; (**g**) **1d** surface of arginine residues with ion intensities for $CH_6N_3^+$, $C_4H_{11}N_2^+$, and $C_4H_{10}N_3^+$; (**h**) **1d** surface of tryptophan residues with ion intensity for $C_9H_8N^+$; (**i**) image of the control Au surface observed at tryptophan-specific ion $C_9H_8N^+$.

ToF-SIMS of series 2 on Au surfaces

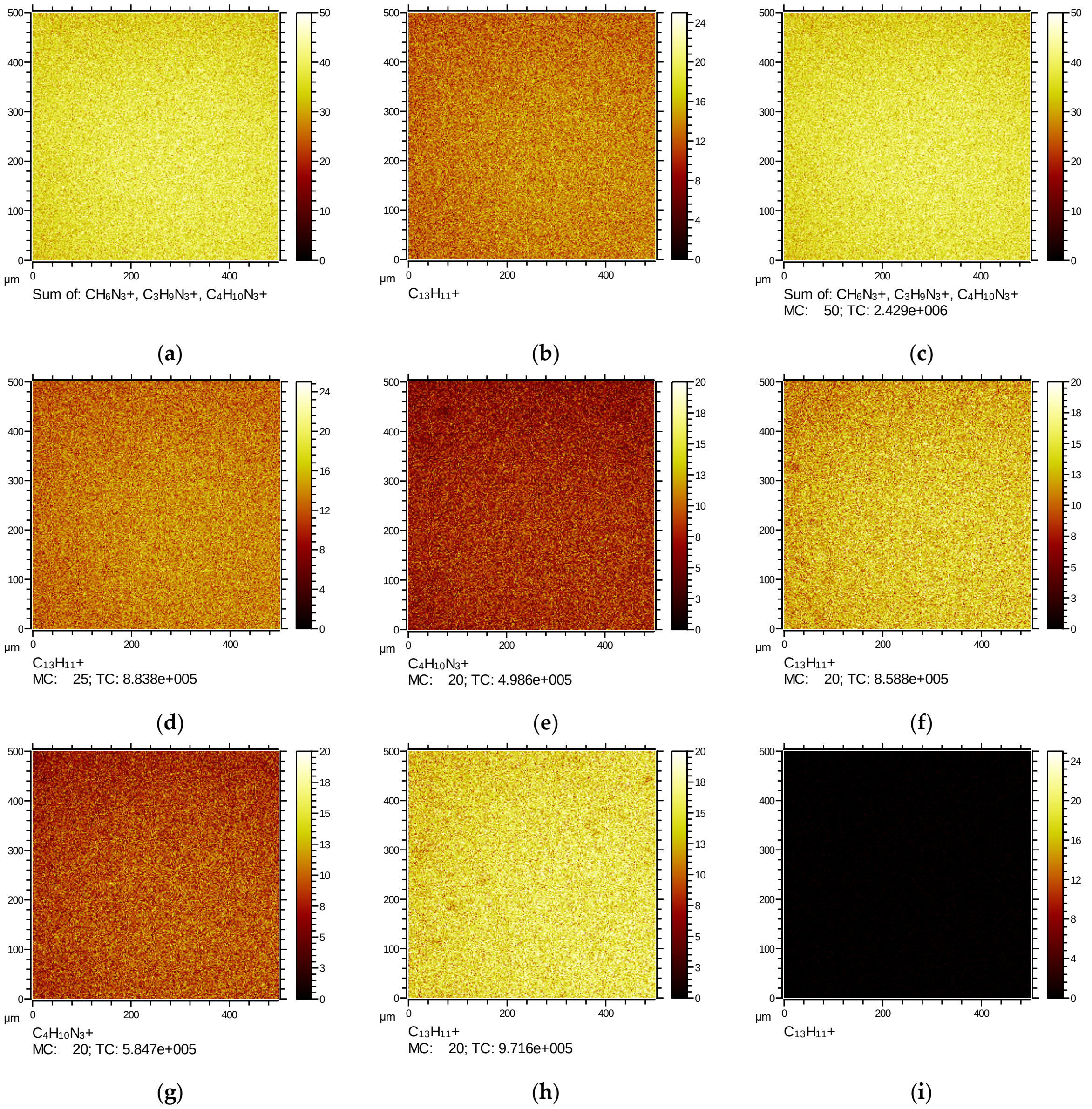

Figure S27. ToF-SIMS images of series 2 containing biphenylalanine: (**a**) **2a** surface of arginine residues with ion intensities for $CH_6N_3^+$, $C_3H_9N_3^+$, and $C_4H_{10}N_3^+$; (**b**) **2a** surface of biphenylalanine residues with ion intensity for $C_{13}H_{11}^+$; (**c**) **2b** surface of arginine residues with ion intensities for $CH_6N_3^+$, $C_3H_9N_3^+$, and $C_4H_{10}N_3^+$; (**d**) **2b** surface of biphenylalanine residues with ion intensity for $C_{13}H_{11}^+$; (**e**) **2c** surface of arginine residues with ion intensity for $C_4H_{10}N_3^+$; (**f**) **2c** surface of biphenylalanine residues with ion intensity for $C_{13}H_{11}^+$; (**g**) **2d** surface of arginine residues with ion intensity for $C_4H_{10}N_3^+$; (**h**) **2d** surface of biphenylalanine residues with ion intensity for $C_{13}H_{11}^+$; (**i**) image of the control Au surface observed at biphenylalanine-specific ion $C_{13}H_{11}^+$.

Certika data

Table S2. Certika data of **1a–d** and **2a–d** covalently linked to Au surface. Au surface without a peptide was used as a control group.

Sample	Replicate #1	Replicate #2	Average surface	Average per surface
Au_1a_1	7.4	7.6	7.5	
Au_1a_2	6.4	6.6	6.5	
Au_1a_3	7.3	7.3	7.3	
Au_1a_4	7.3	7.3	7.3	
Au_1a_5	7.2	7.0	7.1	**7.13**
Au_1b_1	7.9	7.9	7.9	
Au_1b_2	8.1	8.2	8.1	
Au_1b_3	7.4	7.5	7.5	
Au_1b_4	7.1	6.9	7.0	
Au_1b_5	6.8	6.9	6.9	
Au_1b_6	7.5	7.4	7.4	**7.47**
Au_1c_1	8.9	9.0	8.9	
Au_1c_2	6.9	7.1	7.0	
Au_1c_3	6.5	6.5	6.5	
Au_1c_4	6.4	6.3	6.4	
Au_1c_5	6.4	6.5	6.4	**7.03**
Au_1d_1	13.7	17.2	15.4	
Au_1d_2	6.3	6.4	6.4	
Au_1d_3	10.1	10.3	10.2	
Au_1d_4	8.7	8.5	8.6	
Au_1d_5	10.5	10.3	10.4	**10.19**
Au_2a_1	8.7	8.4	8.6	
Au_2a_2	7.9	8.1	8.0	
Au_2a_3	8.9	8.7	8.8	
Au_2a_4	6.9	7.4	7.2	
Au_2a_5	8.7	8.7	8.7	
Au_2a_6	10.9	11.1	11.0	**8.70**
Au_2b_1	14.9	14.3	14.6	
Au_2b_2	7.9	8.2	8.0	
Au_2b_3	8.4	8.5	8,5	
Au_2b_4	11.1	11.0	11.0	
Au_2b_5	11.0	10.7	10.9	
Au_2b_6	10.3	10.5	10.4	**10.60**
Au_2c_1	6.8	6.8	6.8	
Au_2c_2	9.1	9.2	9.2	
Au_2c_3	7.9	7.7	7.8	
Au_2c_4	6.9	6.6	6.7	
Au_2c_5	6.6	6.6	6.6	
Au_2c_6	8.5	8.3	8.4	**7.60**
Au_2d_1	15.6	15.4	15,5	
Au_2d_2	13.8	14.4	14.1	
Au_2d_3	15.0	13.0	14.0	
Au_2d_4	17.9	?	17.9	
Au_2d_5	17.9	17.4	17.7	
Au_2d_6	15.1	14.9	15.0	**15.69**
Au_Control_1	5.8	5.7	5.7	
Au_Control_2	6.1	6.1	6,1	
Au_Control_3	7.1	7.0	7.1	
Au_Control_4	5.5	5.4	5.4	
Au_Control_5	5.5	5.6	5.6	
Au_Control_6	5.6	5.7	5.6	**5.93**

Antimicrobial activity of the peptides

Table S3. Overview of MIC values for **1a–d** and **2a–d** screened against *S. aureus*, *S. epidermidis*, *E. coli*, and *P. aeruginosa*.

		MIC in µg/mL												MIC in µg/mL											
		1a			1b			1c			1d			2a			2b			2c			2d		
**Strain**	**Tech Rep**	**BR1**	**BR2**	**BR3**	**BR1**	**BR2**	**BR3**	**BR1**	**BR2**	**BR3**	**BR1**	**BR2**	**BR3**	**BR1**	**BR2**	**BR3**	**BR1**	**BR2**	**BR3**	**BR1**	**BR2**	**BR3**	**BR1**	**BR2**	**BR3**
S. aureus ATCC 9144	1	64	64	64	128	128	128	32	32	32	8	16	8	8	8	8	16	32	16	8	4	8	8	4	4
	2	64	64	64	128	128	128	32	32	32	8	16	8	8	8	8	16	32	32	8	8	8	4	4	8
	3	64	64	64	128	128	128	32	32	32	8	16	16	8	8	8	16	32	32	8	4	8	4	4	4
S. epidermidis 1457	1	32	32	32	128	128	64	16	16	16	8	8	16	4	8	8	16	16	16	8	4	8	4	2	2
	2	32	32	32	128	128	128	16	16	16	8	8	16	4	8	8	16	16	16	4	4	8	2	2	2
	3	32	32	32	128	128	64	16	16	16	8	8	16	4	8	8	16	16	16	4	4	8	2	2	2
E. coli ATCC 25922	1	128	256	256	>256	>256	>256	32	64	32	64	64	64	64	64	64	128	128	128	8	8	16	64	64	64
	2	128	256	256	>256	>256	>256	64	64	32	64	64	64	64	64	64	128	128	128	8	8	16	64	64	32
	3	128	256	256	>256	>256	>256	64	64	32	64	64	64	64	64	64	128	128	128	8	16	8	64	64	32
P. aeruginosa ATCC 27853	1	256	256	256	>256	>256	>256	64	64	32	128	256	128	64	64	64	128	256	256	8	8	16	256	256	256
	2	256	256	256	>256	>256	>256	64	64	32	128	256	128	64	64	64	128	256	256	16	16	16	256	256	256
	3	256	256	256	>256	>256	>256	64	64	32	128	256	128	64	64	64	128	256	256	16	8	16	256	256	256
